# Entry Steps in the Biosynthetic Pathway to Diterpenoid Alkaloids in *Delphinium grandiflorum* and *Aconitum plicatum*

**DOI:** 10.1101/2025.05.15.654307

**Published:** 2025-05-17

**Authors:** Garret P. Miller, Lana Mutabdžija-Nedelcheva, Trine B. Andersen, Imani Pascoe, Kathryn Van Winkle, Tomáš Pluskal, Björn Hamberger

**Affiliations:** 1 Biochemistry and Molecular Biology, Michigan State University, East Lansing, Michigan, United States; 2 Institute of Organic Chemistry and Biochemistry of the Czech Academy of Sciences, Flemingovo nám. 2, 160 00 Prague, Czechia; 3 Department of Genetics and Microbiology, Charles University, Albertov 6 , 128 00 Prague, Czechia

## Abstract

Roots from the *Aconitum* (Wolf’s-Bane) and *Delphinium* (Larkspur) genera have been widely used in traditional medicine owing to the abundance of bioactive diterpenoid alkaloids that they produce. Despite extensive research on these compounds and their potential medicinal applications, their structural complexity precludes their production through total chemical synthesis, and little progress has been made towards elucidation of their biosynthetic pathways. Here, we report the entry steps in the biosynthesis of the diterpenoid alkaloid atisinium, constituting six enzymes identified from the Siberian Larkspur (*Delphinium grandiflorum*) and garden monkshood (*Aconitum plicatum*) through a combination of comparative transcriptomics between tissue types and genera and coexpression analysis. This pathway includes a pair of terpene synthases, three cytochromes P450, and a reductase with little homology to other characterized enzymes. We further demonstrate, through incorporation of isotopically labelled substrates, the preference of the reductase for ethanolamine over ethylamine, and similarly that ethanolamine is the preferred source of nitrogen for the majority of detected diterpenoid alkaloids. Identification of these enzymes and production of a key intermediate in a heterologous host paves the way for biosynthetic production of this group of metabolites with promise for medicinal applications.

## Introduction

To support their defense, interactions with other organisms, and ecological adaptation, land plants have evolved a plethora of structurally-diverse specialized metabolites. Such metabolites often feature biologically-relevant molecular scaffolds which have proven paramount for the discovery of chemical probes and drugs^1^. Among these, both the alkaloid and terpenoid classes have received considerable attention for their medicinal applications, with prominent examples including taxol^2^ (anti-cancer), artemisinin^3^ (antiplasmodial), morphine^4^ (analgesic), colchicine^5^ (anti-inflammatory), scopolamine^6–8^ (anti-nausea), and vinblastine^9–11^ (anti-cancer). Their importance in the pharmaceutical industry has led to a wealth of research into elucidation of their biosynthetic pathways and production in heterologous hosts, as direct extraction from plants or complete chemical synthesis is often challenging or unfeasible.

Given the unique pathways towards initial scaffold formation, there is little overlap between the terpenoid and alkaloid classes of specialized metabolites. One notable exception is the diterpenoid alkaloids, which are found throughout many independent plant lineages^12–14^, but primarily within the cosmopolitan Ranunculaceae family^15,16^ (Supplementary Fig. 1). The biosynthesis of this class of metabolites has not been elucidated, however it is apparent from their structure that it involves the initial formation of a diterpene scaffold followed by nitrogen incorporation, and extensive modifications by various oxygenases, acyltransferases, and methyltransferases.

**Figure 1:**
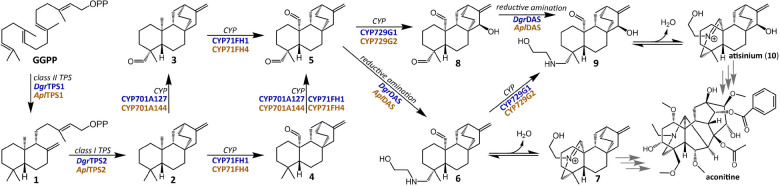
Proposed biosynthetic pathway towards aconitine-type diterpenoid alkaloids. Putative enzyme classes or chemical transformations used to guide our approach are annotated along each arrow, with enzymes characterized in this study for each step from *D. grandiflorum* and *A. plicatum* written in blue and orange, respectively.

Plants from the *Aconitum* and *Delphinium* genera have long been used in traditional medicine due to the bioactivity of diterpenoid alkaloids, which primarily accumulate in their root tissue^17–20^. The use of “Fuzi”— the processed lateral root of *A. carmichaelii* (more commonly known as Wolf’s Bane or Aconite)—has been documented for at least two thousand years^16^. The diterpenoid alkaloids have a wide range of potential applications from antifeedants to anti-cancer, antiplasmodial, cholineesterase inhibitors, and analgesics^15,16,21–23^ with lappaconitine, 3-acetylaconitine and crassicauline A used as non-narcotic analgesic drugs^24^. The therapeutic properties of many of these metabolites have prompted an extensive amount of research into their total chemical synthesis^25–29^, however, their structural complexity presents an enormous challenge in chemical synthesis. This is best exemplified by aconitine (Figure 1), a potent neurotoxin with six interconnected rings and fifteen stereocenters. Despite being first isolated in 1833 by P. L. Geiger and numerous attempts to synthesize it chemically, aconitine has not yet been successfully synthesized by chemists^30^.

Elucidating the biosynthesis of these compounds would ameliorate some of the challenges in their production given the complexity of their scaffolds and the number of required stereospecific oxidations. The lack of current knowledge in their biosynthesis is not for a lack of effort, as there is a large body of work involving transcriptomic analysis of these pathways in various *Aconitum* species^31–35^, with only one case including experimental validation for a pair of terpene synthases^36^ (TPSs) as the first two steps in the pathway. To our knowledge, similar work has not yet been done on the neighboring *Delphinium* genus, despite the likely conservation of this pathway between both genera.

To address the gaps in the biosynthetic pathway of diterpenoid alkaloids, we carried out transcriptome sequencing on *Delphinium grandiflorum*, *Aconitum plicatum* and *Aconitum lycoctonum*, and also included public data from four other *Aconitum* species. Transcriptome assembly both for *D. grandiflorum* and for six representatives of the *Aconitum* species (*A. plicatum, A. lycoctonum, A. carmichaelii, A. japonicum, A. kusnezoffii*, and *A. vilmorinianum*)—all of which accumulate diterpenoid alkaloids—allowed for comparative transcriptomics across tissue types and genera, leading to the identification of six enzymes active in this pathway. Furthermore, the public data for *A. vilmorinianum*—a root tissue timecourse study^31^—allowed for coexpression analysis, resulting in the identification of a novel reductase active in the pathway which has little homology to previously characterized enzymes. This reductase catalyzes a key step in the pathway, supporting the formation of atisinium (**10**, Figure 1)—a bioactive diterpenoid alkaloid and potential intermediate in the biosynthesis of more complex diterpenoid alkaloids^23^. Despite the abundance of diterpenoid alkaloids with ethylamine groups attached to their central terpene scaffolds, we further demonstrate that ethanolamine is the preferred substrate for this reductase and the primary nitrogen source for the majority of detected diterpenoid alkaloids in *Aconitum* plants. Identification of these entry steps will serve as the basis for further pathway discovery towards diterpenoid alkaloid natural products and biosynthetic production in heterologous hosts.

## Results

### A Pair of TPSs Cyclize GGPP to ent-atiserene

The majority of diterpenoid alkaloids in the Ranunculaceae family can be divided into three groups based on the number of carbons in their backbone structure (C_18_, C_19_, and C_20_), each of which is further divided into multiple subgroups represented by 46 distinct scaffolds^15,24^. Despite considerable structural differences between them which reflect variations in their biosynthetic pathways, they appear to share the same initial biosynthesis with the majority of scaffolds believed to arise primarily from *ent*-atiserene^24,37^. Our proposed biosynthetic pathway is detailed in Figure 1. Initially, the cyclization pattern of geranylgeranyl diphosphate (GGPP) follows a class II terpene synthase (TPS) mechanism, with three distinct stereocenters which suggest the involvement of an *ent*-copalyl diphosphate (*ent*-CPP) synthase. The ring pattern of the majority of diterpenoid alkaloids is consistent with that of *ent*-atiserene, suggesting the involvement of a class I TPS to convert *ent*-CPP to this scaffold. Subsequent nitrogen incorporation would first require oxidative functionalization of key methyl groups on the *ent*-atiserene scaffold, likely to aldehydes, suggesting the involvement of one or more cytochrome P450s (CYPs). The final incorporation of this nitrogen group likely involves a reductive amination and marks the transition from diterpenoids to diterpenoid alkaloids^24^.

In order to identify candidate enzymes for this pathway, we opted to cross-reference transcriptomic data across multiple species and tissue types. We isolated and sequenced RNA from several tissues of *Aconitum plicatum* (root, flower, leaf, and stem), *Aconitum lycoctonum* (root, flower, leaf, stem, and fruit), and *Delphinium grandiflorum* (root, leaf, and flower), and carried out *de novo* transcriptome assembly for each. Furthermore, datasets from *A. carmichaelii* (root, leaf, flower, bud; PRJNA415989)^33^, *A. japonicum* (root, root tuber, leaf, flower, stem; PRJDB4889), *A. kusnezoffii* (leaf, principal root, and lateral root; PRJNA670255)^38^ and *A. vilmorinianum* (root time course; PRJNA667080)^31^ from the NCBI Sequence Read Archive (SRA) were included as well.

A BLAST search of the *D. grandiflorum* transcriptome against a reference set of plant TPSs (Supplementary Dataset 1) revealed fourteen putative TPS genes between the two subfamilies typically implicated in diterpene biosynthesis: class II TPSs from the TPS-c subfamily, and class I TPSs from the TPS-e subfamily. Only three of these were exclusively expressed in root tissue, matching the tissue-specific accumulation of diterpenoid alkaloids. *Dgr*TPS1 (TPS-c) and *Dgr*TPS2 (TPS-e) appeared to be the most likely candidates, as they belong to the pair of subfamilies typically implicated in labdane-related diterpene biosynthesis.

**Figure 2:**
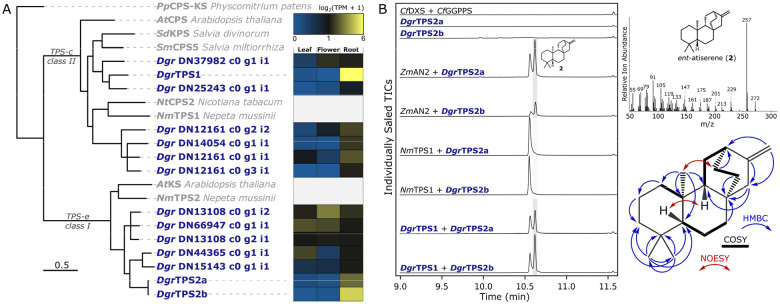
Identification of a pair of terpene synthases which produce ent-atiserene. **A)** Maximum likelihood phylogenetic tree of predicted *D. grandiflorum* TPS sequences and their respective expression across tissue types. Reference TPS sequences are in gray, scale bar represents substitutions per site, and branches with less than 50% bootstrap support have been collapsed. Expanded view of this tree with *Aconitum* sequences is given in Supplementary Fig 2. **B)** GC-MS analysis of *Dgr*TPS1 and *Dgr*TPS2 coexpression in *N. benthamiana*. *Zm*AN2 (*Zea mays*) and *Nm*TPS1 (*Nepeta mussinii*) are *ent*-CPP (**1**) and (+)-CPP (enantiomer of **1**) synthases, respectively. Each isoform of *Dgr*TPS2 selectively converts *ent*-CPP to its product, as indicated by the presence of **2** in the shaded region when paired with an *ent*-CPP (**1**) synthase, but not a (+)-CPP synthase. Marking of 1* indicates the dephosphorylated derivative of **1**, as **1** itself is not detectable by GC-MS. Each assay has *Cf*DXS and *Cf*GGPPS coexpressed in addition to those listed. **C)** Mass spectrum (70 eV EI) of 2 from panel B, and select HMBC, NOESY, and COSY correlations for *ent*-atiserene (**2**) purified from *N. benthamiana* expressing *Dgr*TPS1 and *Dgr*TPS2a.

Full-length genes for *Dgr*TPS1 and *Dgr*TPS2 were cloned from *D. grandiflorum* root cDNA for *Agrobacterium*-mediated transient expression in *Nicotiana benthamiana*. Two isoforms of *Dgr*TPS2, named *Dgr*TPS2a and *Dgr*TPS2b, were cloned from cDNA and both were tested. All screening through transient expression in *N. benthamiana* included coexpression with *Cf*DXS and *Cf*GGPPS (to increase precursor supply of GGPP^38^). GC-MS analysis of hexane extracts revealed that *Dgr*TPS1 acts as a copalyl diphosphate (CPP) synthase. Coexpression of an enantioselective *ent*-kaurene synthase (*Nm*TPS2)^39^ led to production of *ent*-kaurene, suggesting an absolute stereochemistry for the product of *Drg*TPS1 consistent with *ent*-CPP (1) (Figure 2B). Furthermore, *Dgr*TPS2a and 2b showed conversion of **1** to a new product with a fragmentation pattern (Figure 2C) similar to that of *ent*-atiserene (**2**)^40^ for both isoforms. To confirm the identity of this new product as **2**, transient expression in *N. benthamiana* was scaled up with *Dgr*TPS1 and *Dgr*TPS2a, and the product was purified through silica chromatography and confirmed through NMR (Supplementary Table 1 and Supplementary Figure 4). Since both isoforms of *Dgr*TPS2 exhibit the same function, *Dgr*TPS2a was used for further testing and is referred to as *Dgr*TPS2 hereafter. Cloning and characterization of the respective orthologs from *Aconitum plicatum* revealed that both *Apl*TPS1 and *Apl*TPS2 have this same activity and enantioselectivity, and demonstrates the conservation of the entry steps of this pathway across both genera (Supplementary Figure 3).

### Three Cytochrome P450s Oxidize the ent-atiserene scaffold

Following the confirmation that a pair of terpene synthases produce **2**, we continued with our proposed biosynthetic pathway to search for cytochromes P450 (CYPs) which can carry out oxidations of methyl groups 19 and 20 to aldehydes. The proposed intermediate *ent*-atiserene-19-al (**3**) resembles the central metabolite *ent*-kaurenoic acid—a key compound in the primary metabolic pathway towards gibberellins^41^—which is synthesized from GGPP through the activity of a class II/class I TPS pair and a CYP from the CYP701A subfamily^41^. Given the characterization above for a class II/class I TPS pair, it is plausible that the gene responsible for making **3** from **2** is a recent duplicate of a CYP701A, especially given the occurrence of polyploidization within the Delphinieae tribe (containing *Aconitum* and *Delphinium*) of the Ranunculaceae family^42–44^.

In contrast to the TPS family, the identification of CYPs presents a challenge due to the number of genes that may be present in any given plant^45^. In our transcriptome assemblies for *D. grandiflorum* and the six *Aconitum* species, a BLAST search against a reference set of CYP sequences (Supplementary Dataset 1) yielded 2,123 predicted CYP transcripts after clustering by 99% sequence identity, with 284 for *D. grandiflorum* alone and roughly two to four hundred for each *Aconitum* assembly.

**Figure 3:**
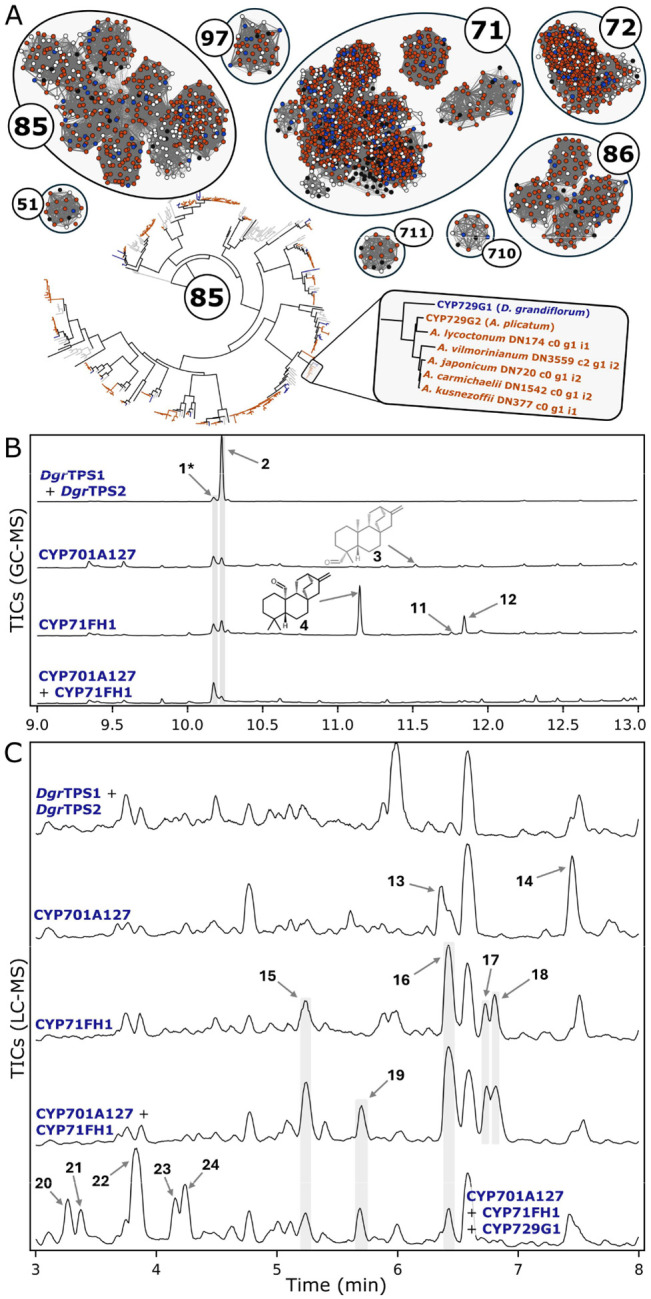
Search strategy and characterization of three CYPs active in the pathway. **A**) Visual representation of the search strategy for candidate CYPs. CYPs mined from each transcriptome were grouped into distinct clans through a sequence similarity network, and each clan was built into an individual phylogenetic tree. Groups of CYPs which match the relative speciation pattern shown in the example–which also had high root expression–were selected for testing. *D. grandiflorum* sequences in blue, *Aconitum* spp. in orange, other Ranunculaceae in white, and reference sequences in black **B**) GC-MS analysis and **C**) LC-MS analysis of CYP candidates expressed in *N. benthamiana*. All assays have *Cf*DXS, *Cf*GGPPS, *Dgr*TPS1, and *Dgr*TPS2 coexpressed in addition to those listed. Mass spectra for all compounds are given in Supplementary Figures 8, 11, and 12.

To narrow this down to a manageable number to test, we took advantage of the assumed conservation of this pathway between neighboring genera and tissue-specific accumulation of metabolites. The total CYP transcripts from each assembly were first assigned to individual clans through a sequence-similarity network, and individual phylogenies were made for distinct clans (Figure 3A and Supplementary Figures 5–7). We then filtered these transcripts to include only those in *D. grandiflorum* with high root expression and with a root-expressed ortholog in each *Aconitum* assembly. This narrowed a list of 284 possible CYPs from *D. grandiflorum* down to just six for testing.

These six CYPs were cloned from *D. grandiflorum* root cDNA and tested through transient expression in *N. benthamiana*. Each candidate was coexpressed with *Dgr*TPS1 and *Dgr*TPS2, and products were analyzed via GC-MS following ethyl acetate extraction. CYP701A127 and CYP71FH1 both showed activity in oxidizing the *ent*-atiserene (**2**) backbone (Figure 3B). Coexpression with either of these CYPs showed a depletion in **2** and the production of respective metabolites with a molecular ion at m/z 286 and retention of m/z 257 as the highest abundance fragment ion (Compounds **3** and **4**; Supplementary Figure 8), consistent with two sequential oxidations of **2** to a carbonyl. CYP71FH1 also produces a major product with a molecular ion at m/z 300 (compound **12**), which would suggest a net addition of two oxygen atoms and four oxidations from **2**.

For the products of CYP71FH1, we scaled up production in *N. benthamiana* to purify compounds and attempt to solve structures by NMR. While sufficient quantities were simple to produce through expression and extraction from roughly 30 g of infiltrated tissue, purification of the two major products from each other proved challenging. One fraction purified through a silica column was sufficiently enriched for the m/z 286 product that we could confirm the identity of **4** as *ent*-atiserene-20-al through NMR (Supplementary Figures 9–10). The structure of the m/z 300 product (**12**) was not determined. The products of CYP701A127 gave weak signals by GC and may have been shuttled away to other products through conversion by endogenous *N. benthamiana* enzymes. We tentatively assigned CYP701A127’s product as *ent*-atiserene-19-al (**3**) based on its similar fragmentation pattern to **4** and phylogenetic placement in the CYP701A subfamily.

In our proposed biosynthetic pathway, we assumed that a pair of CYPs could work together to oxidize both methyl groups at carbons 19 and 20 to aldehydes, and so we tested whether coexpression of both of these enzymes would further the pathway. Coexpression of both CYPs in addition to both TPSs revealed a depletion of both **2** and of both CYPs’ respective products when analyzing ethyl acetate extracts by GC-MS (Figure 3B). These assays were also analyzed by LC-MS on 80% methanol extracts, which revealed two products detectable through coexpression of CYP701A127 (**13** and **14**), four from CYP71FH1 (**15**-**18**), and one additional product with coexpression of both enzymes (**19**) (Figure 3C and Supplemental Figures 11–12). Four of the products present with both CYPs coexpressed are an accumulation of those observed with CYP71FH alone (compounds **15–18**, including its major product **16**), suggesting that these are products different than those detected by GC-MS for CYP71FH1 alone, and that CYP701A127 may share a partial functional redundancy with CYP71FH1.

We further characterized this pair of CYPs against the remaining four candidates. Coexpression of both TPSs, both CYPs, and each remaining CYP candidate revealed that CYP729G1 can oxidize these products (Figures 3C). The molecular ions for each product suggest that they are each a single hydroxylation difference (additional 16 m/z) from major products for CYP701A127 and CYP71FH1 alone.

### Continuation of the Previously Proposed Biosynthetic Pathway

In many alkaloid biosynthetic pathways, the formation of an alkaloid scaffold typically involves the accumulation of both an amine and aldehyde precursor^46^. The nitrogen present in the majority of diterpenoid alkaloids in *Aconitum* and *Delphinium* appears to be derived from ethylamine due to the attached -CH_2_CH_3_ group (e.g. aconitine; Figure 1), while some metabolites presumably incorporate methylamine (-CH_3_) or ethanolamine (-CH_2_CH_2_OH)^15,16^—the origin of which could come from decarboxylation of alanine, glycine, or serine, respectively. Serine decarboxylases are present in primary metabolism, and a duplication of one of these enzymes in *Camellia sinensis* has been shown to decarboxylate alanine into ethylamine (AlaDC) in theanine biosynthesis^47^. Additionally, *Spirea japonica*—an evolutionarily distinct plant which makes similar compounds—has been shown to produce isotopically labelled diterpenoid alkaloids through addition of labelled serine^48^.

**Figure 4:**
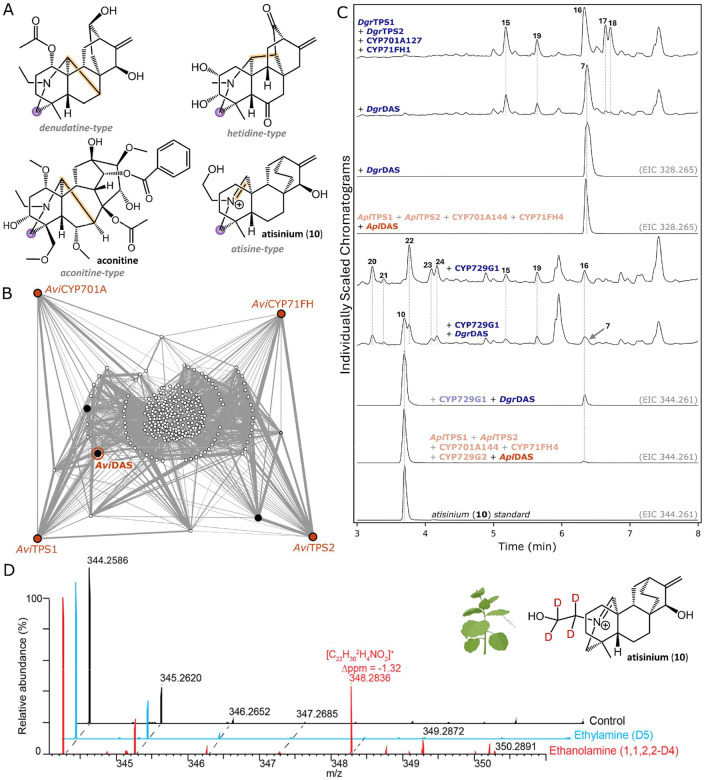
Nitrogen incorporation into diterpenoid alkaloids. **A**) Four example skeleton types of diterpenoid alkaloids. Highlighted in purple is a common attachment point for nitrogen-containing groups that does not typically form an additional carbon-carbon bond, and in yellow are examples of additional carbon-carbon bonds formed on the nitrogen-containing group’s second attachment point, (or a second carbon-nitrogen bond in an unresolved iminium ion in the case of **10**). **B**) Coexpression analysis on *A. vilmorinianum* roots. Four predicted orthologs to the first four pathway genes are drawn as orange nodes, and all genes coexpressed with these four are drawn in a network with nodes representing transcripts and edge width representing magnitude of coexpression between them. Transcripts connected to a greater number of orange nodes are shown further to the outside, with transcripts in the center only connected by two degrees of separation. Drawn in black are three putative reductases which were selected for testing, with DAS highlighted. **C**) LC-MS chromatograms for putative reductase testing in *N. benthamiana*. All genes have *Cf*DXS and *Cf*GGPPS coexpressed in addition to those listed, as well as *Dgr*TPS1, *Dgr*TPS2, CYP701A127, and CYP71FH1, or their respective *A. plicatum* orthologs. *D. grandiflorum* genes are written in blue and respective orthologs of each gene from *A. plicatum* are written in orange. Chromatograms are TICs except where otherwise indicated. **D**) Isotope pattern of atisinium, after coinfiltration of isotopically labelled ethanolamine (1,1,2,2,-D4) and ethylamine (D5) with Agrobacteria carrying atisinium-forming genes from *A. plicatum* in *N. benthamiana*.

The mechanism of nitrogen incorporation is also an important consideration, as the iminium ion formed through condensation of an amine and aldehyde is inherently unstable. Quenching of this cation through either a substitution or reduction^46^ can avoid hydrolysis separating them back into their constituent parts, and in the case of diterpenoid alkaloids, it likely follows both mechanisms based on the number of carbon-carbon bonds present on both attachment points for the nitrogen-containing group (Figure 4A). Carbon 20 almost always contains an extra carbon-carbon bond relative to **2** and the intermediate **4**, while carbon 19 does not, similar to both **2** and the putative intermediate **3**. This suggests that incorporation at carbon 19 requires a reductase, and at carbon 20 may involve a spontaneous intramolecular condensation.

In contrast to the steps elucidated thus far, involving carbocation-mediated cyclizations (TPSs) and site-specific oxidations (CYPs), the reaction of an amine and aldehyde to form an alkaloid scaffold could occur either spontaneously or through enzyme catalysis given the inherent reactivity between aldehydes and primary amines. The search for a putative reductase is also not straightforward when considering how many different enzyme families from which this function could evolve. To search for the next step(s), we carried out a coexpression analysis to see which genes were coexpressed with the first four enzymes already characterized in the pathway (*Dgr*TPS1, *Dgr*TPS2, CYP701A127, and CYP71FH1). Given that our RNA sequencing only contained single replicates of each tissue type, we carried out this analysis on data collected for *A. vilmorinianum* instead (Figure 4B), which involved sequencing three replicates of root tissue at three different stages of development^31^. Among the genes found to be highly coexpressed with the *A. vilmorinianum* orthologs of our first four pathway genes, three were identified as putative reductases, and so the respective orthologs from *D. grandiflorum* and *A. plicatum* were selected for characterization.

### Coexpression Analysis Reveals that a Predicted Reductase is Active in the Pathway

Each of these three putative reductases were cloned from *D. grandiflorum* root cDNA and tested for activity through transient expression in *N. benthamiana* and LC-MS analysis on 80% methanol extracts. Testing of each candidate was carried out along with either the first four pathway enzymes (*Dgr*TPS1, *Dgr*TPS2, CYP701A127, and CYP71FH1) or these four plus CYP729G1. In addition, we cloned every respective *A. plicatum* ortholog of each CYP listed above and included these in testing for the same combinations.

Coexpression of *Dgr*DAS (diterpenoid alkaloid synthase) with only the first four pathway enzymes led to a depletion in previously observed products and the formation of a new peak **7** with with a proposed neutral formula of C_22_H_33_NO (exact mass 328.2656 in ESI+; calculated m/z 328.2640: Figure 4C and Supplementary Figure 13). Coexpression of *Dgr*DAS with the first four pathway enzymes and CYP729G1 did not deplete all of CYP729G1’s products, however did lead to the formation of a new peak **10** which was identified as atisinium (Figure 4C and Supplementary Figure 15), a diterpenoid alkaloid with antiplasmodial activity^23^ and a potential intermediate in the biosynthetic pathway of downstream diterpenoid alkaloids. To confirm the conservation of this biosynthetic step between both genera, the *A. plicatum* DAS ortholog (*Apl*DAS) was cloned and characterized, which also resulted in atisinium (**10**) formation upon coexpression with *A. plicatum’s* respective orthologs for preceding pathway genes (Figure 4C).

Although the nitrogen in **10** appears to be derived from ethanolamine, many diterpenoid alkaloids contain an ethylamine group. We carried out an initial test to determine the source of nitrogen through coexpression of an alanine decarboxylase from *C. sinensis* (AlaDC)^48^, which would presumably increase the supply of ethylamine *in planta*. We observed very little change in product profile upon coexpression of this enzyme with preceding pathway enzymes except for trace amounts of one additional peak per combination (Supplementary Figure 13). We therefore sought to test the substrate specificity of *Apl*DAS to assess its potential involvement in formation of various scaffolds that serve as branching points in diterpenoid alkaloid biosynthetic pathways. For that, in addition to the heterologous expression of characterized genes in *N. benthamiana*, we co-infiltrated either isotopically labelled ethanolamine (1,1,2,2-D_4_) or ethylamine (D_5_). Atisinium (**10**) isotopic peak distribution demonstrated the incorporation of labelled ethanolamine over ethylamine, showing a clear preference of *Apl*DAS for ethanolamine (Figure 4D). This raises the question whether or not additional reductases are present in the DA pathway thus leading to different scaffolds, and if ethanolamine is the preferred source of nitrogen in *Aconitum* and *Delphinium* plants.

### Ethanolamine as a preferred source of nitrogen in Aconitum diterpenoid alkaloids

**Figure 5:**
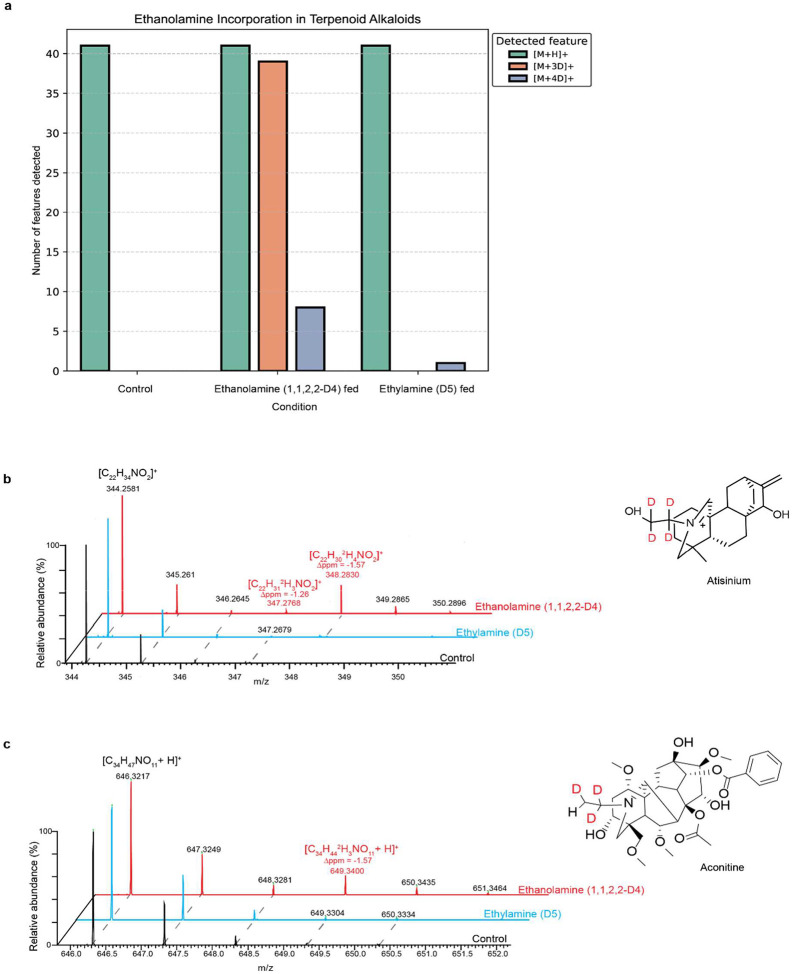
Incorporation of deuterium-labelled compounds in callus culture **a)** LC-MS features classified as “Terpenoid alkaloids” by CANOPUS across feeding conditions for which M+3D and M+4D isotopologues were detected, highlighting ethanolamine as the preferred source of nitrogen **b)** isotope pattern of atisinium, highlighting the isotopologue peaks corresponding to incorporation of three and four deuteriums **c)** isotope pattern of aconitine, highlighting the isotopologue peak corresponding to incorporation of ethanolamine with three deuteriums.

In order to better understand the biosynthesis of diterpenoid alkaloids and particularly the source of nitrogen, we developed callus cultures of *A. plicatum* and fed them with either isotopically labelled ethanolamine (1,1,2,2-D4), ethylamine (D5) or 1 x PBS (control) over the course of one month. Due to the scarcity of available standards and lack of diterpenoid alkaloids in spectral libraries, we opted for computational solutions, in order to obtain a list of all detected and predicted diterpenoid alkaloids from callus LC-MS analysis (see Supplementary Fig. 16 for the computational workflow). Computational solutions for metabolite annotation problems in untargeted metabolomics have seen an expansion in recent years, with SIRIUS^49^ being one of the most prominent. It relies on isotope patterns and fragmentation trees for molecular formula prediction, with further steps involving formula ranking (ZODIAC^50^), prediction of a molecular fingerprint of the query compound through CSI:FingerID^51^ and compound class prediction (CANOPUS^52^). Relying on this solution, out of 1,331 features with MS/MS spectra collected across all the samples, 144 were classified by SIRIUS as belonging to terpenoid alkaloids. Filtering only for features that were consistently present across the three feeding conditions, defined as being detected in at least one replicate of each condition, as well as the ones whose classification had a probability score higher than 0.6 further reduced the list to 60 putative terpenoid alkaloids. An additional feature was added manually, namely atisinium (**10**), which the computational pipeline failed to classify as a terpenoid alkaloid, thus resulting in a final table with 61 detected features. Interestingly, out of those, 41 features show the incorporation of isotopically labelled ethanolamine (Figure 5a), whereas only atisinium showed incorporation of both ethanolamine and ethylamine (Figure 5b). However, taking into account that the detected isotopologue corresponds to incorporation of four deuteriums, and that it shows a very low intensity, this might be due to the fact that ethylamine in callus was oxidized to ethanolamine, and subsequently incorporated.

Out of 41 features with ethanolamine incorporation, two features included only the isotopologue with four deuteriums detected, while six features showed detection of both three deuteriums and four deuteriums. The remaining features had only the three-deuterium isotopologue detected, with the loss of one deuterium possibly explained by deuterium-hydrogen exchange resulting from enzymatic activity^53^. Despite ethylamine being intuitively inferred as the source of nitrogen, given its presence as the side group in the majority of diterpenoid alkaloids (aconitine included, Figure 5c), no incorporation was observed. This could be attributed to the computational pipeline used to identify terpenoid alkaloids, which may overlook some and thus lead to their exclusion from the analysis. The difference in the number of detected diterpenoid alkaloids compared to the number of diterpenoid alkaloids with incorporated labelled substrate can be attributed to the fact that some diterpenoid alkaloids are being stored rather than actively synthesised by the calli during feeding, as well as the potential utilization of an alternative nitrogen source. Furthermore, the detection of secreted labelled diterpenoid alkaloid compounds in the agar could suggest their role in plant-plant communication, which has been poorly investigated so far^54^.

## Discussion

Through a combination of comparative transcriptomics and coexpression analysis, we have identified six enzymes active in the biosynthetic pathway towards diterpenoid alkaloids conserved between the *Delphinium* and *Aconitum* genera within the Ranunculaceae family. There are hundreds of diterpenoid alkaloids in this family, and the identification of these enzymes will serve as the basis for further pathway discovery towards specific metabolites. This work highlights the utility of cross-referencing transcriptomic data between genera as an orthogonal filter for selection of candidate enzymes beyond the analysis of a single species, as it likely would not have been possible to identify all of these enzymes otherwise given the inherent complexity of these pathways.

Following our characterization of the TPS pair which produce *ent*-atiserene (2), Mao et al. 2021^36^ published a characterization of the entire TPS family from *A. carmichaelli* and identified enzymes orthologous to ours. Many preceding studies have focused on this biosynthetic pathway,^31–35^, and Mao et al.^36^ commented that this work can be complicated by incomplete transcript assembly of genes putatively annotated as being involved in the pathway, and as such combined two different methods of RNA sequencing (Illumina and PacBio) to assemble their transcriptome. In fact, in our initial assembly for *D. grandiflorum*, transcripts for *Dgr*TPS1 and *Dgr*TPS2 were truncated. While we didn’t troubleshoot why our assembly failed to assemble them correctly the first time, we were confident that these candidates were correct based on their expression pattern and phylogenetic origin, and we simply reassembled the transcriptome with a limited dataset (see Methods) and both genes were assembled properly.

One possible explanation for these assembly artifacts is that the genetics of members of the *Delphinium* and *Aconitum* genera are inherently complicated. *Delphinium montanum*, for example, is an autotetraploid with a predicted genome size of roughly 40 Gb^44^ (2n = 32^55^). The seven species studied here have a range of predicted ploidy levels (*D. grandiflorum*: 2n = 16; *A. carmichaelii*: 2n = 32/64 – depending on cultivar; *A. japonicum*: 2n = 32; *A. vilmorinianum*: 2n = 16; *A. kusnezoffii*: 2n=32; *A. plicatum*: 2n=32; *A. lycoctonum*: *2n*=*16*)^55–57^, and it has been suggested that, at least in the *Aconitum* genus, there may have been multiple recent events of polyploidization and diploidization^43^. This fits with the model of our initial biosynthetic pathway—and the phylogenetic relationships of these genes—in which we predicted that the first three steps may be recent duplications of primary metabolism enzymes given the similarity of these predicted intermediates to those in gibberellin biosynthesis^41^. While we didn’t characterize the putative central metabolism copies of these genes, Mao et al.^36^ characterized a pair of recently-duplicated *ent*-CPP synthases and *ent*-kaurene/atiserene synthases in their analysis. CYP701A127 (from *D. grandiflorum*) and CYP701A144 (from *A. plicatum*), which we tentatively assigned as *ent*-atiserene (**2**) oxidases that forms *ent*-atiserene-19-al (**3**), also belongs to the same subfamily as CYP701A3, the *ent*-kaurene oxidase involved in gibberellin metabolism in *Arabidopsis*^58^.

It should be noted that *Dgr*TPS1 and *Apl*TPS1—being *ent*-CPP (**1**) synthases—are technically not enzymes that produce specialized metabolites. Given their high and exclusive expression in roots relative to their putative central metabolism paralogs, however, they are likely dedicated to specialized metabolism. A similar phenomenon is seen in both *Oryza sativa*^59^ and *Zea mays*^60^, where two copies of an *ent*-CPP synthase are present; one which is involved in gibberellin biosynthesis and another which is inducible by pathogens for the production of defensive *ent*-CPP-derived specialized metabolites. Given the presence of duplicate *ent*-CPP synthases in each of these independent lineages of plants, there is likely a strong evolutionary pressure for the ability to tightly regulate these competing pathways.

Throughout the process, we varied the approach to identify each class of enzyme based on what information was necessary. For the TPSs, for example, few enough transcripts were present in our assembly that we relied solely on data from *D. grandiflorum*. For the CYPs, cross-referencing both *Delphinium* and *Aconitum* datasets was essential given the presence of two to three hundred unique transcripts in each assembly. Choosing to work with two neighboring genera allowed us to filter these hundreds of candidates down to just six, as the only orthologous genes present across each species have persisted over roughly 27 million years since the speciation between the two genera^61^. Finally, even with tissue and species-specific transcriptomic data, the following step for nitrogen incorporation was not obvious; therefore, coexpression analysis allowed us to search for new candidates without prior knowledge of which enzyme families to search.

Throughout the process of characterizing various steps in the pathway, not every intermediate product was identified prior to moving forward with following steps. Often it can be difficult to differentiate “actual” intermediates in terms of whether the observed products are relevant to the pathway or simply a result of an incomplete reconstruction or a heterologous host’s interference of the native pathway. In the process of discovering the biosynthetic pathway for the diterpenoid forskolin from *Coleus forskohlii*, for example, coexpression of an incomplete set of genes in *N. benthamiana* led to an accumulation of many side products that were not present once the entire pathway was reconstructed (five CYPs acting on a single diterpene scaffold and at least sixteen total products)^62^. A similar–but deconstructive–example can be seen with accumulation of precursors and side products for the scopolamine pathway in *Atropa belladonna* following virus-induced gene silencing of various pathway steps^6^. This may be reflective of the ability for specialized metabolism to evolve towards a range of different products with only a small change (e.g. a single gene deletion) in their respective biosynthetic pathways. We identified the activity of the two TPSs and confirmed our predicted activity of the first two CYPs, but thereafter we decided to test enzymes in different combinations to identify new steps in case the side products seen were due to a similar phenomenon. Given the appearance of multiple products with the first four pathway enzymes and convergence to primarily a single product **7** upon coexpression with *Dgr*DAS or *Apl*DAS, the initial range of products is likely a result of incomplete pathway reconstruction.

We initially proposed that ethylamine was the source of nitrogen in this pathway due to the abundance of diterpenoid alkaloids that have been identified with an ethyl group bonded to the nitrogen. The presence of a minor product forming upon coexpression with AlaDC was expected based on the presence of aldehydes in our intermediates, however the amount of product that would form was uncertain. We demonstrate here through isotope feeding studies that the preferred substrate for *Apl*DAS is ethanolamine, and that diterpenoid alkaloids generally incorporate ethanolamine preferentially over ethylamine, even in cases like aconitine where an ethylene group is present in the final product. It remains to be seen how this is implicated in further biosynthetic steps towards more complex diterpenoid alkaloids.

Beyond the immediate questions emerging from this work, more questions remain in the discovery of diterpenoid alkaloid pathways. Perhaps the most important is the differentiation between the C20 and C19/C18 metabolites, and at which point this occurs. The greatest challenge will likely be the reconstruction of an entire pathway to a specific final product, rather than the initial scaffold-forming steps investigated here, which are presumably common to all diterpenoid alkaloids. Based on the structure of aconitine (Figure 1), there are potentially twenty or more enzymatic steps involved in its biosynthesis. Further pathway discovery of such downstream steps will likely require different methodology than employed here given the species-specificity of some of these products. This may benefit from accurate cross-species metabolomic data to differentiate chemical conversions present in distinct lineages in conjunction with the cross-species transcriptomics employed here.

## Materials and Methods

### Plant material, RNA isolation, and cDNA synthesis

*D. grandiflorum* plants were grown in a greenhouse under ambient photoperiod and 24°C day/17°C night temperatures. RNA isolation, quality assessment, RNA sequencing, and cDNA synthesis for *D. grandiflorum* was carried out as described in Miller et al. 2020^63^. Total RNA was isolated from flowers, leaves, and roots with the Spectrum Plant Total RNA Kit (Sigma-Aldrich), and DNA was removed with the DNA-free DNA Removal Kit (Thermo Fisher Scientific). Quality of the resulting RNA was assessed with a Qubit (Thermo Fisher Scientific) and RNA-nano assays (Agilent Bioanalyzer 2100). RNA sequencing was carried out on an Illumina HiSeq 4000 at Novogene (Sacramento, CA, USA) *Aconitum plicatum* and *Aconitum lycoctonum* plants were collected from the Charles University Botanical Garden. For these species, RNA was isolated from roots, flowers, leaves, stems, and (for *A. lycoctonum*) fruits using the RNeasy kit (Qiagen) according to the manufacturer’s instructions. The transcriptome library preparation and sequencing were performed at the Beijing Genomics Institute using the DNBSEQ Eukaryotic Strand-specific mRNA library.

### D. grandiflorum and Aconitum spp. de novo transcriptome assembly and analysis

RNA-seq data were obtained through RNA sequencing on an Illumina HiSeq 4000 for *D. grandiflorum*, and from DNBseq platform for *A. plicatum* and *A. lycoctonum*. Raw data are accessible on the NCBI Sequence Read Archive (SRA; ncbi.nlm.nih.gov/sra) under the accession PRJNA1261909. Additional data for *A. carmichaelii* (PRJNA415989)^33^, *A. japonicum* (PRJDB4889), *A. kusnezoffii* (PRJNA670255), and *A. vilmorinianum* (PRJNA667080)^31^ were obtained from the SRA. Reads were filtered for quality by a Phred score of 10 or higher and adapters trimmed with TrimGalore (v0.6.10; github.com/FelixKrueger/TrimGalore). Unless otherwise specified, a maximum of 150 million read pairs (or single-end reads) were used for each transcriptome assembly, and were taken evenly across samples.

Transcriptome assembly and analysis followed a similar pipeline as is described in Miller et al. 2020^63^. Transcriptomes were assembled *de novo* with Trinity (v2.8.5)^64^ and open reading frames and translations were found with TransDecoder (v5.7.1)^65^. Transcriptomes were clustered at 99% sequence identity by local alignment to at least 15% of the larger sequence with CD-HIT (v4.8.1)^66,67^, and any sequences filtered out by this step were also filtered out from the translated TransDecoder output. Expression levels were calculated with Salmon (v0.14.1)^68^ with the transcriptome filtered by CD-HIT used as the index. The results of this analysis are accessible at doi.org/10.5281/zenodo.15384649, and a Nextflow pipeline which automates this process can be found at github.com/GarretPMiller/DiterpenoidAlkaloids.

Initial assembly of the *D. grandiflorum* transcriptome resulted in incomplete transcripts for *Dgr*TPS1 and *Dgr*TPS2 (only ~75% coverage of reference sequences), and although this was prior to our characterization of these enzymes, we noted that these transcripts were most likely misassembled given their high expression and likelihood of being involved in the pathway.

Reassembly of the *D. grandiflorum* transcriptome was therefore done with only data acquired from root tissue, with reads from each tissue type mapped to this assembly. Transcripts for both of these genes in the new assembly aligned to the entire length of reference sequences, and so this assembly was used for further analysis.

### Sequence Similarity Network and Phylogenetic Trees

Sequences from all transcriptome assemblies, predicted gene models from two other Ranunculaceae species (*Thalictrum thalictroides*: PRJNA439007; *Coptis chinensis*: PRJNA662860), and reference sequences (given in Supplemental Dataset 1) were used to build the sequence similarity network and all phylogenetic trees. CYP sequence similarity network was made with BLAST (v2.7.1+)^69^ at a sequence identity threshold of 42% and visualized with Cytoscape^70^. Sequences which only had one connection (primarily fungal contaminants and low percent identity reference sequences) were removed from the network. Multiple sequence alignments used for phylogenetic tree construction were made with Clustal Omega (v1.2.4)^71^, and maximum likelihood phylogenetic trees were made with RAxML (v8.2.12)^72^. All trees were the result of 1,000 bootstrap replicates, except where otherwise indicated. Branches with less than 50% bootstrap support were collapsed in Figure 2, but are left uncollapsed with bootstrap values indicated at nodes for all other trees. Vector images of all trees used in this study are accessible at doi.org/10.5281/zenodo.15384649.

### Coexpression analysis

Our assembly for *A. vilmorinianum* was used for coexpression analysis. To minimize the computational burden, we reduced the analysis through clustering by 99% identity with CD-HIT (v4.8.1)^66,67^, calculated expression levels through mapping reads to this clustered transcriptome, and eliminated any transcript with no samples that had at least 20% the expression level (in TPM) as any sample for either TPS. Coexpression analysis was carried out as described by Wisecaver et al. 2017^73^ (pipeline at: https://github.itap.purdue.edu/jwisecav/mr2mods). The resulting coexpression network shown in Figure 4B shows only genes with one or two degrees of separation from any of the first four genes in the pathway (respective orthologs from *A. vilmorinianum* of *Dgr*TPS1/*Apl*TPS1, *Dgr*TPS2/*Apl*TPS2, CYP701A127/CYP701A144, and CYP71FH1/CYP71FH4) based on a mutual rank (MR) cutoff of ê(-(MR-1)/5) > 0.01.

### Cloning

Candidate genes were PCR-amplified from root cDNA and cloned into pEAQ-HT^74^ through In-Fusion cloning. Constructs for *Zm*AN2, *Nm*TPS1, and *Nm*TPS2 in pEAQ (used as positive controls for *ent*-CPP, (+)-CPP, and *ent*-kaurene biosynthesis, respectively) were made by Johnson et al. 2019^39^. Nucleotide (verified by Sanger sequencing) and amino acid sequences for all *Delphinium* and *Aconitum* genes tested here are given in Supplemental Dataset 1.

### Transient expression in N. benthamiana, product scale-up, and NMR analysis

Transient expression in *N. benthamiana* for screening assays was carried out as described in Miller et al. 2020^63^, with the exception of solvents used to extract each set of assays as described in the main text. For the co-infiltration of ethanolamine (1,1,2,2-D4) and ethylamine (D5), the compounds were dissolved in distilled water, filter sterilized, and co-infiltrated in *N. benthamiana* leaves 1 day after the candidate genes were infiltrated. For *ent*-atiserene and *ent*-atiserene-20-al scaleup, three whole plants were infiltrated with a syringe, and approximately 15/30 g of fresh weight were extracted with hexane/ethyl acetate (respectively). Products were purified through silica chromatography with 10% ethyl acetate : 90% hexane as the mobile phase. Initial purification was carried out with approximately 100 mL of oven-dried silica, and fractions were collected in approximately 3 mL increments and assessed for purity by GC-FID. Fractions containing desired products were further purified with approximately 1.5 mL oven-dried silica in a Pasteur pipette, with fractions collected in 1 mL increments and purity assessed by GC-FID. NMR analysis was carried out on a Bruker 800 MHz spectrometer equipped with a TCl cryoprobe using CDCl_3_ as the solvent. CDCl_3_ peaks were referenced to 7.26 and 77.00 ppm for ^1^H and ^13^C spectra, respectively.

### GC-MS analysis

All GC-MS analyses were performed on hexane or ethyl acetate extracts (described for each case in the text) with an Agilent 7890A GC with an Agilent VF-5ms column (30 m x 250 μm x 0.25 μm, with 10m EZ-Guard) and an Agilent 5975C mass spectrometer. The inlet was set to 250°C splitless injection of 1 μL, He carrier gas (1 ml/min), and the detector was activated following a 3 min solvent delay. Mass spectra were generated using 70 eV electron ionization with a scan range of m/z 50 to 350. The following method was used for analysis of each sample presented in the text: temperature ramp start 40°C, hold 1 min, 40°C/min to 200°C, hold 2 min, 20°C/min to 280°C, 40°C/min to 320°C; hold 5 min. For initial GC-MS analysis of *Apl*TPS1 and *Apl*TPS2 products shown in Supplementary Figure 3, the GC method varied by the following parameters: 275°C splitless injection, scan range 50 to 400 m/z, 4.5 minute hold after reaching 200°C, 20°C/min to 240°C, 10°C/min to 280°C. Figures for chromatograms and mass spectra were generated with Pyplot.

### LC-MS analysis

All LC-MS analyses (except as specified below) were performed on 80% methanol : 20% H_2_O *N. benthamiana* extracts with a Waters Xevo G2-XS quadrupole ToF mass spectrometer with a Waters ACQUITY column manager and Waters ACQUITY BEH C18 column (2.1 × 100 mm; 1.7 μm). Injection volume for each sample was 10 μL, and flow rate was set to 0.3 mL/min with a column temperature of 40°C. The mobile phase consisted of 10 mM ammonium formate (pH 2.8) (Solvent A) and acetonitrile (Solvent B) with the following method: initial 99% A : 1 % B , continuous gradient to 2% A : 98% B over 12 min, hold for 1.5 min, continuous gradient to 99% A : 1% B over 0.1 min, hold 1.5 min. Mass spectra were generated through electrospray ionization in positive-ion mode with leucine enkephalin as a lockmass, and continuum peak acquisition were collected with a mass range of m/z 50–1500 and a scan duration of 0.2 s. Capillary and cone voltage were 3.0 kV and 40 V, respectively, cone and desolvation gas flow rates were 40 and 600 L/h, respectively, and source and desolvation temperatures were 100°C and 350°C, respectively. High-energy spectra were generated with argon as the collision gas and a voltage ramp from 20 to 80 V. Figures for chromatograms and mass spectra were generated with Pyplot.

LC-MS analysis of isotopically labelled atisinium were performed on 80% MeOH extracts of *N. benthamiana* with a Vanquish Flex UHPLC system interfaced to an Orbitrap ID-X Tribrid mass spectrometer using heated electrospray ionization (H-ESI). For LC analysis, Waters ACQUITY BEH C18 column (2.1 × 150mm, x 2.1; 1.7 μm) was used. Injection per sample was 1uL, with flow rate of 0.35 mL/min with the column temperature of 40°C. The mobile phase consisted of water with 0.1% formic acid (Solvent A) and acetonitrile with 0.1% formic acid (Solvent B) with the following method: initial 95% A : 5 % B, continuous gradient to 100% B over 15.5 min, hold for 1.8 min, and a continuous gradient to 95% A over 2 minutes. Mass spectra were generated through H-ESI in positive mode, and continuum peak acquisition were collected with a mass range of m/z 100–1000. Capillary voltage was at 3.0 kV, ion transfer tube temperature was 325°C, auxilary gass flow rate 10 L/min, vaporizer temperature 350 **°C**, sheath gas flow rate 50 L/min, MS resolution 60,000, RF Lens 45% and maximum injection time 118 ms. The same method was used for the analysis of callus cultures, except the resolution was increased to 120,000 to improve the distinction of isotopic peaks arising as a result of deuterium incorporation.

### Callus culture establishment, substrate feeding and metabolite extraction

*A. plicatum* petioles were cut into 0.5cm pieces and sterilized by immersion in 70% ethanol over 2 minutes, followed by 20 minutes in 20% bleach (sodium hypochlorite) and rinsed with sterile distilled water. The sterilized petioles were placed on ½ Murashige and Skoog (MS) medium (Duchefa Biochemie, M0222) supplemented with 0.8% agar (Sigma-Aldrich, A7921), 3% sucrose, 0.1 mg/L kinetin and 1 mg/L 1-Naphthaleneacetic acid (NAA). Cultures were incubated in darkness at room temperature until callus formation was observed. Established calli were then transferred to fresh medium and maintained under the same conditions.

For the substrate feeding studies, calli were divided into three groups: (1) ethanolamine (1,1,2,2-D4)-fed (Cambridge Isotopes Laboratories, DLM-552–0.1), (2) ethylamine (D5)-fed (Cambridge Isotopes Laboratories, DLM-3471–0.5), and (3) control group to which 1xPBS was added. A sterile 1mM solution of each substrate was applied directly on the callus surface, biweekly over the course of one month. During this period, calli were kept in darkness at room temperature. For the metabolite extraction, calli were collected in 2 mL Safe-Lock Eppendorf tubes, weighed, and extracted with ethyl acetate at a 1:10 (w/v) ratio. Samples were homogenized using a steel bead in a Qiagen TissueLyser. After centrifugation at 18,000 x g, the supernatant was collected and transferred to LC-MS vials.

### Computational metabolomics analysis of callus cultures

Raw LC-MS data files were converted to open format (.mzML) using MSConvert^75^ tool from ProteoWizard^76^ package. Untargeted feature detection was performed in MZmine 3^77^ using the mzmine configuration batch file. This generated a feature list where each detected isotopic peak was retained as an individual feature. Additionally, an alternative feature list was created using a different batch file for analysis with SIRIUS software (v6.0.7). Default parameters were used except for the instrument type, which was set to Orbitrap. Molecular formula prediction was done based on isotope pattern and fragmentation tree reconstruction, with formula ranking refined using ZODIAC. Feature properties and compound class were computed using CSI:FingerID module and CANOPUS, respectively. Jupyter Notebook was used to filter SIRIUS results, creating a database of all compounds classified as terpenoid alkaloids. This database was then used to identify terpenoid alkaloid features that exhibited incorporation of labelled deuteriums. The used script, as well as the batch files and the final output table can be freely accessed at https://github.com/lalalana5/Callus-culture-isotope-labelling/tree/main. Each predicted compound with incorporated deuterium was manually verified by inspecting the raw data.

## Supplementary Material

Supplement 1

1

## Data Availability

RNA sequencing data for D. grandiflorum, A. plicatum, and A. lycoctonum can be found at the NCBI Sequence Read Archive under the BioProject accession number PRJNA1261909. Nucleotide sequences verified by Sanger sequencing for each characterized gene are submitted to GenBank: (GenBank accessions pending). These sequences, along with amino acid translations and all amino acid sequences of reference TPSs and CYPs used to build the phylogenetic trees can be found in Supplemental Dataset 1. Transcriptome assemblies, open reading frames, and expression values across RNA-seq samples are provided in the following Zenodo repository: doi.org/10.5281/zenodo.15384649. All phylogenetic trees are also available in this repository (doi.org/10.5281/zenodo.15384649). A Nextflow pipeline to carry out the transcriptome assembly and analysis process done here can be found at: github.com/GarretPMiller/DiterpenoidAlkaloids. The script, batch files, and final output table for the computational metabolomics of callus cultures can be found at: github.com/lalalana5/Callus-culture-isotope-labelling/tree/main.

## References

[1] Li F.-S.; Weng J.-K. Demystifying Traditional Herbal Medicine with Modern Approach. Nat. Plants 2017, 3 (8), 1–7. 10.1038/nplants.2017.109. 28758992

[2] Alternative sources and metabolic engineering of Taxol: Advances and future perspectives - ScienceDirect. https://www.sciencedirect.com/science/article/pii/S0734975020300665?casa_token=iBcjS8xspLYAAAAA:APTC3Qe63H7BKmM53FMDx2xfMN6pJ_-F-SQOx61BvQYgYXstDi0Xh0fkNQi3kT1s41LYuAnhNyI (accessed 2025–01-02). 10.1016/j.biotechadv.2020.107569 32446923

[3] Huang Y.; Yang Y.; Liu G.; Xu M. New Clinical Application Prospects of Artemisinin and Its Derivatives: A Scoping Review. Infect. Dis. Poverty 2023, 12 (1), 115. 10.1186/s40249-023-01152-6. 38072951 PMC10712159

[4] Galanie S.; Thodey K.; Trenchard I. J.; Filsinger Interrante M.; Smolke C. D. Complete Biosynthesis of Opioids in Yeast. Science 2015, 349 (6252), 1095–1100. 10.1126/science.aac9373. 26272907 PMC4924617

[5] Nett R. S.; Lau W.; Sattely E. S. Discovery and Engineering of Colchicine Alkaloid Biosynthesis. Nature 2020, 584 (7819), 148–153. 10.1038/s41586-020-2546-8. 32699417 PMC7958869

[6] Bedewitz M. A.; Jones A. D.; D’Auria J. C.; Barry C. S. Tropinone Synthesis via an Atypical Polyketide Synthase and P450-Mediated Cyclization. Nat. Commun. 2018, 9, 5281. 10.1038/s41467-018-07671-3. 30538251 PMC6290073

[7] Wrenbeck E. E.; Bedewitz M. A.; Klesmith J. R.; Noshin S.; Barry C. S.; Whitehead T. A. An Automated Data-Driven Pipeline for Improving Heterologous Enzyme Expression. ACS Synth. Biol. 2019, 8 (3), 474–481. 10.1021/acssynbio.8b00486. 30721031 PMC6855305

[8] Biosynthesis of medicinal tropane alkaloids in yeast | Nature. https://www.nature.com/articles/s41586-020-2650-9 (accessed 2024–12-05). 10.1038/s41586-020-2650-9 PMC7529995 32879484

[9] Pan Q.; Mustafa N. R.; Tang K.; Choi Y. H.; Verpoorte R. Monoterpenoid Indole Alkaloids Biosynthesis and Its Regulation in Catharanthus Roseus: A Literature Review from Genes to Metabolites. Phytochem. Rev. 2016, 15 (2), 221–250. 10.1007/s11101-015-9406-4.

[10] Caputi L.; Franke J.; Farrow S. C.; Chung K.; Payne R. M. E.; Nguyen T.-D.; Dang T.-T. T.; Soares Teto Carqueijeiro I.; Koudounas K.; Dugé de Bernonville T.; Ameyaw B.; Jones D. M.; Vieira I. J. C.; Courdavault V.; O’Connor S. E. Missing Enzymes in the Biosynthesis of the Anticancer Drug Vinblastine in Madagascar Periwinkle. Science 2018, 360 (6394), 1235–1239. 10.1126/science.aat4100. 29724909

[11] Qu Y.; Safonova O.; De Luca V. Completion of the Canonical Pathway for Assembly of Anticancer Drugs Vincristine/Vinblastine in Catharanthus Roseus. Plant J. 2019, 97 (2), 257–266. 10.1111/tpj.14111. 30256480

[12] The alkaloids of species of Garrya. I. Isolation of alkaloids - ScienceDirect. https://www.sciencedirect.com/science/article/abs/pii/S0095955315310581?via%3Dihub (accessed 2024–12-05).

[13] Ma Y.; Mao X.-Y.; Huang L.-J.; Fan Y.-M.; Gu W.; Yan C.; Huang T.; Zhang J.-X.; Yuan C.-M.; Hao X.-J. Diterpene Alkaloids and Diterpenes from Spiraea Japonica and Their Anti-Tobacco Mosaic Virus Activity. Fitoterapia 2016, 109, 8–13. 10.1016/j.fitote.2015.11.019. 26625838

[14] Hart N.; Johns S.; Lamberton J.; Suares H.; Willing R. New Alkaloids of the Ent-Kaurene Type From Anopterus Species (Escalloniaceae). I. The Structure and Reactions of Anopterine. Aust. J. Chem. 1976, 29 (6), 1295–1318. 10.1071/ch9761295.

[15] Yin T.; Cai L.; Ding Z. An Overview of the Chemical Constituents from the Genus Delphinium Reported in the Last Four Decades. RSC Adv. 2020, 10 (23), 13669–13686. 10.1039/D0RA00813C. 35492993 PMC9051563

[16] Nyirimigabo E.; Xu Y.; Li Y.; Wang Y.; Agyemang K.; Zhang Y. A Review on Phytochemistry, Pharmacology and Toxicology Studies of Aconitum. J. Pharm. Pharmacol. 2015, 67 (1), 1–19. 10.1111/JPHP.12310. 25244533

[17] Xu J.-B.; Li Y.-Z.; Huang S.; Chen L.; Luo Y.-Y.; Gao F.; Zhou X.-L. Diterpenoid Alkaloids from the Whole Herb of Delphinium Grandiflorum L. Phytochemistry 2021, 190, 112866. 10.1016/j.phytochem.2021.112866. 34271299

[18] Li Y.; Gao F.; Zhang J.-F.; Zhou X.-L. Four New Diterpenoid Alkaloids from the Roots of Aconitum Carmichaelii. Chem. Biodivers. 2018, 15 (7), e1800147. 10.1002/cbdv.201800147. 29785743

[19] Yamashita H.; Takeda K.; Haraguchi M.; Abe Y.; Kuwahara N.; Suzuki S.; Terui A.; Masaka T.; Munakata N.; Uchida M.; Nunokawa M.; Kaneda K.; Goto M.; Lee K.-H.; Wada K. Four New Diterpenoid Alkaloids from Aconitum Japonicum Subsp. Subcuneatum. J. Nat. Med. 2018, 72 (1), 230–237. 10.1007/s11418-017-1139-9. 29052027

[20] Yin T.-P.; Cai L.; Fang H.-X.; Fang Y.-S.; Li Z.-J.; Ding Z.-T. Diterpenoid Alkaloids from Aconitum Vilmorinianum. Phytochemistry 2015, 116, 314–319. 10.1016/j.phytochem.2015.05.002. 26021734

[21] Csupor D.; Wenzig E. M.; Zupkó I.; Wölkart K.; Hohmann J.; Bauer R. Qualitative and Quantitative Analysis of Aconitine-Type and Lipo-Alkaloids of Aconitum Carmichaelii Roots. J. Chromatogr. A 2009, 1216 (11), 2079–2086. 10.1016/j.chroma.2008.10.082. 19019379

[22] Zhou G.; Tang L.; Zhou X.; Wang T.; Kou Z.; Wang Z. A Review on Phytochemistry and Pharmacological Activities of the Processed Lateral Root of Aconitum Carmichaelii Debeaux. J. Ethnopharmacol. 2015, 160, 173–193. 10.1016/j.jep.2014.11.043. 25479152

[23] Wangchuk P.; Bremner J. B.; Samten; Skelton B. W.; White A. H.; Rattanajak R.; Kamchonwongpaisan S. Antiplasmodial Activity of Atisinium Chloride from the Bhutanese Medicinal Plant, Aconitum Orochryseum. J. Ethnopharmacol. 2010, 130 (3), 559–562. 10.1016/j.jep.2010.05.057. 20561926

[24] Shen Y.; Liang W. J.; Shi Y. N.; Kennelly E. J.; Zhao D. K. Structural Diversity, Bioactivities, and Biosynthesis of Natural Diterpenoid Alkaloids†. Nat. Prod. Rep. 2020, 37 (6), 763–796. 10.1039/d0np00002g. 32129397

[25] Liu X.-Y.; Wang F.-P.; Qin Y. Synthesis of Three-Dimensionally Fascinating Diterpenoid Alkaloids and Related Diterpenes. Acc. Chem. Res. 2021, 54 (1), 22–34. 10.1021/acs.accounts.0c00720. 33351595

[26] Gong J.; Chen H.; Liu X.-Y.; Wang Z.-X.; Nie W.; Qin Y. Total Synthesis of Atropurpuran. Nat. Commun. 2016, 7 (1), 12183. 10.1038/ncomms12183. 27387707 PMC4941107

[27] Owens K. R.; McCowen S. V.; Blackford K. A.; Ueno S.; Hirooka Y.; Weber M.; Sarpong R. Total Synthesis of the Diterpenoid Alkaloid Arcutinidine Using a Strategy Inspired by Chemical Network Analysis. J. Am. Chem. Soc. 2019, 141 (35), 13713–13717. 10.1021/jacs.9b05815. 31276621 PMC7771644

[28] Pang L.; Liu C.-Y.; Gong G.-H.; Quan Z.-S. Synthesis, in Vitro and in Vivo Biological Evaluation of Novel Lappaconitine Derivatives as Potential Anti-Inflammatory Agents. Acta Pharm. Sin. B 2020, 10 (4), 628–645. 10.1016/j.apsb.2019.09.002. 32322467 PMC7161710

[29] Cherney E. C.; Baran P. S. Terpenoid-Alkaloids: Their Biosynthetic Twist of Fate and Total Synthesis. Isr. J. Chem. 2011, 51 (3–4), 391–405. 10.1002/ijch.201100005. 26207071 PMC4508874

[30] Zhou R.-J.; Dai G.-Y.; Zhou X.-H.; Zhang M.-J.; Wu P.-Z.; Zhang D.; Song H.; Liu X.-Y.; Qin Y. Progress towards the Synthesis of Aconitine: Construction of the AE Fragment and Attempts to Access the Pentacyclic Core. Org. Chem. Front. 2019, 6 (3), 377–382. 10.1039/C8QO01228H.

[31] Li Y.-G.; Mou F.-J.; Li K.-Z. De Novo RNA Sequencing and Analysis Reveal the Putative Genes Involved in Diterpenoid Biosynthesis in Aconitum Vilmorinianum Roots. 3 Biotech 2021, 11 (2), 96. 10.1007/s13205-021-02646-6. PMC7840826 33520582

[32] Pal T.; Malhotra N.; Chanumolu S. K.; Chauhan R. S. Next-Generation Sequencing (NGS) Transcriptomes Reveal Association of Multiple Genes and Pathways Contributing to Secondary Metabolites Accumulation in Tuberous Roots of Aconitum Heterophyllum Wall. Planta 2015, 242 (1), 239–258. 10.1007/S00425-015-2304-6/FIGURES/11. 25904478

[33] Rai M.; Rai A.; Kawano N.; Yoshimatsu K.; Takahashi H.; Suzuki H.; Kawahara N.; Saito K.; Yamazaki M. De Novo RNA Sequencing and Expression Analysis of Aconitum Carmichaelii to Analyze Key Genes Involved in the Biosynthesis of Diterpene Alkaloids. Mol. J. Synth. Chem. Nat. Prod. Chem. 2017, 22 (12). 10.3390/MOLECULES22122155. PMC6150021 29206203

[34] Yang Y.; Hu P.; Zhou X.; Wu P.; Si X.; Lu B.; Zhu Y.; Xia Y. Transcriptome Analysis of Aconitum Carmichaelii and Exploration of the Salsolinol Biosynthetic Pathway. Fitoterapia 2020, 140. 10.1016/J.FITOTE.2019.104412. 31698060

[35] Zhao D.; Shen Y.; Shi Y.; Shi X.; Qiao Q.; Zi S.; Zhao E.; Yu D.; Kennelly E. J. Probing the Transcriptome of Aconitum Carmichaelii Reveals the Candidate Genes Associated with the Biosynthesis of the Toxic Aconitine-Type C19-Diterpenoid Alkaloids. Phytochemistry 2018, 152, 113–124. 10.1016/j.phytochem.2018.04.022. 29758520

[36] Mao L.; Jin B.; Chen L.; Tian M.; Ma R.; Yin B.; Zhang H.; Guo J.; Tang J.; Chen T.; Lai C.; Cui G.; Huang L. Functional Identification of the Terpene Synthase Family Involved in Diterpenoid Alkaloids Biosynthesis in Aconitum Carmichaelii. Acta Pharm. Sin. B 2021. 10.1016/J.APSB.2021.04.008. PMC8546855 34729318

[37] Hong Y. J.; Tantillo D. J. Formation of Beyerene, Kaurene, Trachylobane, and Atiserene Diterpenes by Rearrangements That Avoid Secondary Carbocations. J. Am. Chem. Soc. 2010, 132 (15), 5375–5386. 10.1021/ja9084786. 20353180

[38] Andersen-Ranberg J.; Kongstad K. T.; Nielsen M. T.; Jensen N. B.; Pateraki I.; Bach S. S.; Hamberger B.; Zerbe P.; Staerk D.; Bohlmann J.; Møller B. L.; Hamberger B. Expanding the Landscape of Diterpene Structural Diversity through Stereochemically Controlled Combinatorial Biosynthesis. Angew. Chem. - Int. Ed. 2016, 55 (6), 2142–2146. 10.1002/anie.201510650. PMC4755150 26749264

[39] Johnson S. R.; Bhat W. W.; Bibik J.; Turmo A.; Hamberger B.; Hamberger B. A Database-Driven Approach Identifies Additional Diterpene Synthase Activities in the Mint Family (Lamiaceae). J. Biol. Chem. 2019, 294 (4), 1349–1362. 10.1074/jbc.RA118.006025. 30498089 PMC6349103

[40] Jin B.; Cui G.; Guo J.; Tang J.; Duan L.; Lin H.; Shen Y.; Chen T.; Zhang H.; Huang L. Functional Diversification of Kaurene Synthase-Like Genes in Isodon Rubescens. Plant Physiol. 2017, 174 (2), 943–955. 10.1104/pp.17.00202. 28381502 PMC5462038

[41] Grennan A. K. Gibberellin Metabolism Enzymes in Rice. Plant Physiol. 2006, 141 (2), 524–526. 10.1104/pp.104.900192. 16760495 PMC1475483

[42] Kong H.; Zhang Y.; Hong Y.; Barker M. S. Multilocus Phylogenetic Reconstruction Informing Polyploid Relationships of Aconitum Subgenus Lycoctonum (Ranunculaceae) in China. Plant Syst. Evol. 2017, 303 (6), 727–744. 10.1007/s00606-017-1406-y.

[43] Park S.; An B.; Park S. Recurrent Gene Duplication in the Angiosperm Tribe Delphinieae (Ranunculaceae) Inferred from Intracellular Gene Transfer Events and Heteroplasmic Mutations in the Plastid matK Gene. Sci. Rep. 2020, 10 (1), 2720. 10.1038/s41598-020-59547-6. 32066766 PMC7026143

[44] Salvado P.; Aymerich Boixader P.; Parera J.; Vila Bonfill A.; Martin M.; Quélennec C.; Lewin J.-M.; Delorme-Hinoux V.; Bertrand J. A. M. Little Hope for the Polyploid Endemic Pyrenean Larkspur (Delphinium Montanum): Evidences from Population Genomics and Ecological Niche Modeling. Ecol. Evol. 2022, 12 (3), e8711. 10.1002/ece3.8711. 35342590 PMC8932081

[45] Nelson D.; Werck-Reichhart D. A P450-Centric View of Plant Evolution. Plant J. 2011, 66 (1), 194–211. 10.1111/j.1365-313X.2011.04529.x. 21443632

[46] Lichman B. R. The Scaffold-Forming Steps of Plant Alkaloid Biosynthesis. Nat. Prod. Rep. 2021, 38 (1), 103–129. 10.1039/D0NP00031K. 32745157

[47] Bai P.; Wang L.; Wei K.; Ruan L.; Wu L.; He M.; Ni D.; Cheng H. Biochemical Characterization of Specific Alanine Decarboxylase (AlaDC) and Its Ancestral Enzyme Serine Decarboxylase (SDC) in Tea Plants (Camellia Sinensis). BMC Biotechnol. 2021, 21 (1), 17. 10.1186/s12896-021-00674-x. 33648478 PMC7923638

[48] Zhao P.-J.; Gao S.; Fan L.-M.; Nie J.-L.; He H.-P.; Zeng Y.; Shen Y.-M.; Hao X.-J. Approach to the Biosynthesis of Atisine-Type Diterpenoid Alkaloids. J. Nat. Prod. 2009, 72 (4), 645–649. 10.1021/np800657j. 19275222

[49] Dührkop K.; Fleischauer M.; Ludwig M.; Aksenov A. A.; Melnik A. V.; Meusel M.; Dorrestein P. C.; Rousu J.; Böcker S. SIRIUS 4: A Rapid Tool for Turning Tandem Mass Spectra into Metabolite Structure Information. Nat. Methods 2019, 16 (4), 299–302. 10.1038/s41592-019-0344-8. 30886413

[50] Ludwig M.; Nothias L.-F.; Dührkop K.; Koester I.; Fleischauer M.; Hoffmann M. A.; Petras D.; Vargas F.; Morsy M.; Aluwihare L.; Dorrestein P. C.; Böcker S. Database-Independent Molecular Formula Annotation Using Gibbs Sampling through ZODIAC. Nat. Mach. Intell. 2020, 2 (10), 629–641. 10.1038/s42256-020-00234-6.

[51] Dührkop K.; Shen H.; Meusel M.; Rousu J.; Böcker S. Searching Molecular Structure Databases with Tandem Mass Spectra Using CSI:FingerID. Proc. Natl. Acad. Sci. 2015, 112 (41), 12580–12585. 10.1073/pnas.1509788112. 26392543 PMC4611636

[52] Dührkop K.; Nothias L.-F.; Fleischauer M.; Reher R.; Ludwig M.; Hoffmann M. A.; Petras D.; Gerwick W. H.; Rousu J.; Dorrestein P. C.; Böcker S. Systematic Classification of Unknown Metabolites Using High-Resolution Fragmentation Mass Spectra. Nat. Biotechnol. 2021, 39 (4), 462–471. 10.1038/s41587-020-0740-8. 33230292

[53] Schmidt H.-L.; Werner R. A.; Eisenreich W. Systematics of 2H Patterns in Natural Compounds and Its Importance for the Elucidation of Biosynthetic Pathways. Phytochem. Rev. 2003, 2 (1–2), 61–85. 10.1023/B:PHYT.0000004185.92648.ae.

[54] Waller G. R.; Burström H. Diterpenoid Alkaloids as Plant Growth Inhibitors. Nature 1969, 222 (5193), 576–578. 10.1038/222576a0.

[55] Bosch i Daniel M.; Simon Pallisé J.; López i Pujol J.; Blanché i Vergés C. DCDB: An Updated on-Line Database of Chromosome Numbers of Tribe Delphinieae (Ranunculaceae). 2016.

[56] Mitka J.; Sutkowska A.; Ilnicki T.; Joachimiak A. Reticulate Evolution of High-Alpine Aconitum (Ranunculaceae) in the Eastern Sudetes and Western Carpathians (Central Europe). Acta Biol. Cracoviensia Ser. Bot. 2007, 49, 15–26.

[57] Molero J.; Rovira A. M.; Bosch M.; Simon J.; Blanché C. Karyological Study of the Genus Aconitum (Ranunculacae) in the W Mediterranean Area. Flora Mediterr. 2016, 26, 229–239. 10.7320/FlMedit26.229.

[58] Morrone D.; Chen X.; Coates R. M.; Peters R. J. Characterization of the Kaurene Oxidase CYP701A3, a Multifunctional Cytochrome P450 from Gibberellin Biosynthesis. Biochem. J. 2010, 431 (3), 337–347. 10.1042/BJ20100597. 20698828

[59] Prisic S.; Xu M.; Wilderman P. R.; Peters R. J. Rice Contains Two Disparate Ent-Copalyl Diphosphate Synthases with Distinct Metabolic Functions. Plant Physiol. 2004, 136 (4), 4228–4236. 10.1104/pp.104.050567. 15542489 PMC535852

[60] Harris L. J.; Saparno A.; Johnston A.; Prisic S.; Xu M.; Allard S.; Kathiresan A.; Ouellet T.; Peters R. J. The Maize An2 Gene Is Induced by Fusarium Attack and Encodesan Ent-Copalyl Diphosphate Synthase. Plant Mol. Biol. 2005, 59 (6), 881–894. 10.1007/s11103-005-1674-8. 16307364

[61] Kumar S.; Stecher G.; Suleski M.; Hedges S. B. TimeTree: A Resource for Timelines, Timetrees, and Divergence Times. Mol. Biol. Evol. 2017, 34 (7), 1812–1819. 10.1093/molbev/msx116. 28387841

[62] Pateraki I.; Andersen-Ranberg J.; Jensen N. B.; Wubshet S. G.; Heskes A. M.; Forman V.; Hallström B.; Hamberger B.; Motawia M. S.; Olsen C. E.; Staerk D.; Hansen J.; Møller B. L.; Hamberger B. Total Biosynthesis of the Cyclic AMP Booster Forskolin from Coleus Forskohlii. eLife 2017, 6, e23001. 10.7554/eLife.23001. 28290983 PMC5388535

[63] Miller G. P.; Bhat W. W.; Lanier E. R.; Johnson S. R.; Mathieu D. T.; Hamberger B. The Biosynthesis of the Anti-Microbial Diterpenoid Leubethanol in Leucophyllum Frutescens Proceeds via an All-Cis Prenyl Intermediate. Plant J. 2020, 104 (3), 693–705. 10.1111/tpj.14957. 32777127 PMC7649979

[64] Grabherr M. G.; Haas B. J.; Yassour M.; Levin J. Z.; Thompson D. A.; Amit I.; Adiconis X.; Fan L.; Raychowdhury R.; Zeng Q.; Chen Z.; Mauceli E.; Hacohen N.; Gnirke A.; Rhind N.; di Palma F.; Birren B. W.; Nusbaum C.; Lindblad-Toh K.; Friedman N.; Regev A. Full-Length Transcriptome Assembly from RNA-Seq Data without a Reference Genome. Nat. Biotechnol. 2011, 29 (7), 644–652. 10.1038/nbt.1883. 21572440 PMC3571712

[65] Haas B. J. TransDecoder, 2025. https://github.com/TransDecoder/TransDecoder.

[66] Li W.; Godzik A. Cd-Hit: A Fast Program for Clustering and Comparing Large Sets of Protein or Nucleotide Sequences. Bioinformatics 2006, 22 (13), 1658–1659. 10.1093/bioinformatics/btl158. 16731699

[67] Fu L.; Niu B.; Zhu Z.; Wu S.; Li W. CD-HIT: Accelerated for Clustering the next-Generation Sequencing Data. Bioinformatics 2012, 28 (23), 3150–3152. 10.1093/bioinformatics/bts565. 23060610 PMC3516142

[68] Patro R.; Duggal G.; Love M. I.; Irizarry R. A.; Kingsford C. Salmon Provides Fast and Bias-Aware Quantification of Transcript Expression. Nat. Methods 2017, 14 (4), 417–419. 10.1038/nmeth.4197. 28263959 PMC5600148

[69] Altschul S. F.; Gish W.; Miller W.; Myers E. W.; Lipman D. J. Basic Local Alignment Search Tool. J. Mol. Biol. 1990, 215 (3), 403–410. 10.1016/S0022-2836(05)80360-2. 2231712

[70] Shannon P.; Markiel A.; Ozier O.; Baliga N. S.; Wang J. T.; Ramage D.; Amin N.; Schwikowski B.; Ideker T. Cytoscape: A Software Environment for Integrated Models of Biomolecular Interaction Networks. Genome Res. 2003, 13 (11), 2498–2504. 10.1101/gr.1239303. 14597658 PMC403769

[71] Sievers F.; Wilm A.; Dineen D.; Gibson T. J.; Karplus K.; Li W.; Lopez R.; McWilliam H.; Remmert M.; Söding J.; Thompson J. D.; Higgins D. G. Fast, Scalable Generation of High-quality Protein Multiple Sequence Alignments Using Clustal Omega. Mol. Syst. Biol. 2011, 7 (1), 539. 10.1038/msb.2011.75. 21988835 PMC3261699

[72] Stamatakis A. RAxML Version 8: A Tool for Phylogenetic Analysis and Post-Analysis of Large Phylogenies. Bioinformatics 2014, 30 (9), 1312–1313. 10.1093/bioinformatics/btu033. 24451623 PMC3998144

[73] Wisecaver J. H.; Borowsky A. T.; Tzin V.; Jander G.; Kliebenstein D. J.; Rokas A. A Global Coexpression Network Approach for Connecting Genes to Specialized Metabolic Pathways in Plants. Plant Cell 2017, 29 (5), 944–959. 10.1105/TPC.17.00009. 28408660 PMC5466033

[74] Sainsbury F.; Thuenemann E. C.; Lomonossoff G. P. pEAQ: Versatile Expression Vectors for Easy and Quick Transient Expression of Heterologous Proteins in Plants. Plant Biotechnol. J. 2009, 7 (7), 682–693. 10.1111/j.1467-7652.2009.00434.x. 19627561

[75] Adusumilli R.; Mallick P. Data Conversion with ProteoWizard msConvert. In Proteomics: Methods and Protocols; Comai L., Katz J. E., Mallick P., Eds.; Springer: New York, NY, 2017; pp 339–368. 10.1007/978-1-4939-6747-6_23. 28188540

[76] Chambers M. C.; Maclean B.; Burke R.; Amodei D.; Ruderman D. L.; Neumann S.; Gatto L.; Fischer B.; Pratt B.; Egertson J.; Hoff K.; Kessner D.; Tasman N.; Shulman N.; Frewen B.; Baker T. A.; Brusniak M.-Y.; Paulse C.; Creasy D.; Flashner L.; Kani K.; Moulding C.; Seymour S. L.; Nuwaysir L. M.; Lefebvre B.; Kuhlmann F.; Roark J.; Rainer P.; Detlev S.; Hemenway T.; Huhmer A.; Langridge J.; Connolly B.; Chadick T.; Holly K.; Eckels J.; Deutsch E. W.; Moritz R. L.; Katz J. E.; Agus D. B.; MacCoss M.; Tabb D. L.; Mallick P. A Cross-Platform Toolkit for Mass Spectrometry and Proteomics. Nat. Biotechnol. 2012, 30 (10), 918–920. 10.1038/nbt.2377. 23051804 PMC3471674

[77] Integrative analysis of multimodal mass spectrometry data in MZmine 3 | Nature Biotechnology. https://www.nature.com/articles/s41587-023-01690-2 (accessed 2025–01-23). 10.1038/s41587-023-01690-2 PMC10496610 36859716

[78] Zuntini A. R.; Carruthers T.; Maurin O.; Bailey P. C.; Leempoel K.; Brewer G. E.; Epitawalage N.; Françoso E.; Gallego-Paramo B.; McGinnie C.; Negrão R.; Roy S. R.; Simpson L.; Toledo Romero E.; Barber V. M. A.; Botigué L.; Clarkson J. J.; Cowan R. S.; Dodsworth S.; Johnson M. G.; Kim J. T.; Pokorny L.; Wickett N. J.; Antar G. M.; DeBolt L.; Gutierrez K.; Hendriks K. P.; Hoewener A.; Hu A.-Q.; Joyce E. M.; Kikuchi I. A. B. S.; Larridon I.; Larson D. A.; De Lírio E. J.; Liu J.-X.; Malakasi P.; Przelomska N. A. S.; Shah T.; Viruel J.; Allnutt T. R.; Ameka G. K.; Andrew R. L.; Appelhans M. S.; Arista M.; Ariza M. J.; Arroyo J.; Arthan W.; Bachelier J. B.; Bailey C. D.; Barnes H. F.; Barrett M. D.; Barrett R. L.; Bayer R. J.; Bayly M. J.; Biffin E.; Biggs N.; Birch J. L.; Bogarín D.; Borosova R.; Bowles A. M. C.; Boyce P. C.; Bramley G. L. C.; Briggs M.; Broadhurst L.; Brown G. K.; Bruhl J. J.; Bruneau A.; Buerki S.; Burns E.; Byrne M.; Cable S.; Calladine A.; Callmander M. W.; Cano Á.; Cantrill D. J.; Cardinal-McTeague W. M.; Carlsen M. M.; Carruthers A. J. A.; De Castro Mateo A.; Chase M. W.; Chatrou L. W.; Cheek M.; Chen S.; Christenhusz M. J. M.; Christin P.-A.; Clements M. A.; Coffey S. C.; Conran J. G.; Cornejo X.; Couvreur T. L. P.; Cowie I. D.; Csiba L.; Darbyshire I.; Davidse G.; Davies N. M. J.; Davis A. P.; Van Dijk K.; Downie S. R.; Duretto M. F.; Duvall M. R.; Edwards S. L.; Eggli U.; Erkens R. H. J.; Escudero M.; De La Estrella M.; Fabriani F.; Fay M. F.; Ferreira P. D. L.; Ficinski S. Z.; Fowler R. M.; Frisby S.; Fu L.; Fulcher T.; Galbany-Casals M.; Gardner E. M.; German D. A.; Giaretta A.; Gibernau M.; Gillespie L. J.; González C. C.; Goyder D. J.; Graham S. W.; Grall A.; Green L.; Gunn B. F.; Gutiérrez D. G.; Hackel J.; Haevermans T.; Haigh A.; Hall J. C.; Hall T.; Harrison M. J.; Hatt S. A.; Hidalgo O.; Hodkinson T. R.; Holmes G. D.; Hopkins H. C. F.; Jackson C. J.; James S. A.; Jobson R. W.; Kadereit G.; Kahandawala I. M.; Kainulainen K.; Kato M.; Kellogg E. A.; King G. J.; Klejevskaja B.; Klitgaard B. B.; Klopper R. R.; Knapp S.; Koch M. A.; Leebens-Mack J. H.; Lens F.; Leon C. J.; Léveillé-Bourret É.; Lewis G. P.; Li D.-Z.; Li L.; Liede-Schumann S.; Livshultz T.; Lorence D.; Lu M.; Lu-Irving P.; Luber J.; Lucas E. J.; Luján M.; Lum M.; Macfarlane T. D.; Magdalena C.; Mansano V. F.; Masters L. E.; Mayo S. J.; McColl K.; McDonnell A. J.; McDougall A. E.; McLay T. G. B.; McPherson H.; Meneses R. I.; Merckx V. S. F. T.; Michelangeli F. A.; Mitchell J. D.; Monro A. K.; Moore M. J.; Mueller T. L.; Mummenhoff K.; Munzinger J.; Muriel P.; Murphy D. J.; Nargar K.; Nauheimer L.; Nge F. J.; Nyffeler R.; Orejuela A.; Ortiz E. M.; Palazzesi L.; Peixoto A. L.; Pell S. K.; Pellicer J.; Penneys D. S.; Perez-Escobar O. A.; Persson C.; Pignal M.; Pillon Y.; Pirani J. R.; Plunkett G. M.; Powell R. F.; Prance G. T.; Puglisi C.; Qin M.; Rabeler R. K.; Rees P. E. J.; Renner M.; Roalson E. H.; Rodda M.; Rogers Z. S.; Rokni S.; Rutishauser R.; De Salas M. F.; Schaefer H.; Schley R. J.; Schmidt-Lebuhn A.; Shapcott A.; Al-Shehbaz I.; Shepherd K. A.; Simmons M. P.; Simões A. O.; Simões A. R. G.; Siros M.; Smidt E. C.; Smith J. F.; Snow N.; Soltis D. E.; Soltis P. S.; Soreng R. J.; Sothers C. A.; Starr J. R.; Stevens P. F.; Straub S. C. K.; Struwe L.; Taylor J. M.; Telford I. R. H.; Thornhill A. H.; Tooth I.; Trias-Blasi A.; Udovicic F.; Utteridge T. M. A.; Del Valle J. C.; Verboom G. A.; Vonow H. P.; Vorontsova M. S.; De Vos J. M.; Al-Wattar N.; Waycott M.; Welker C. A. D.; White A. J.; Wieringa J. J.; Williamson L. T.; Wilson T. C.; Wong S. Y.; Woods L. A.; Woods R.; Worboys S.; Xanthos M.; Yang Y.; Zhang Y.-X.; Zhou M.-Y.; Zmarzty S.; Zuloaga F. O.; Antonelli A.; Bellot S.; Crayn D. M.; Grace O. M.; Kersey P. J.; Leitch I. J.; Sauquet H.; Smith S. A.; Eiserhardt W. L.; Forest F.; Baker W. J. Phylogenomics and the Rise of the Angiosperms. Nature 2024, 629 (8013), 843–850. 10.1038/s41586-024-07324-0. 38658746 PMC11111409

