# Supplementary material for: Entry Steps in the Biosynthetic Pathway to Diterpenoid Alkaloids in Delphinium grandiflorum and Aconitum plicatum
Source: bioRxiv. 2025 May 17:2025.05.15.654307. Preprint. [Version 1] doi: 10.1101/2025.05.15.654307 (PMC12132485; doi:10.1101/2025.05.15.654307)
Supplement: 1 [file NIHPP2025.05.15.654307V1-supplement-1.pdf]

bioRxiv preprint doi: https://doi.org/10.1101/2025.05.15.654307; this version posted May 17, 2025. The copyright holder for this preprint (which was not certified by peer review) is the author/funder, who has granted bioRxiv a license to display the preprint in perpetuity. It is made available under aCC-BY 4.0 International license.

Supplementary Information for:

Entry Steps in the Biosynthetic Pathway to Diterpenoid Alkaloids in *Delphinium grandiflorum* and *Aconitum plicatum*

Garret P. Miller*, Lana Mutabdžija-Nedelcheva*2, Trine B. Andersen, Imani Pascoe, Kathryn Van Winkle, Tomáš Pluskal, Björn Hamberger

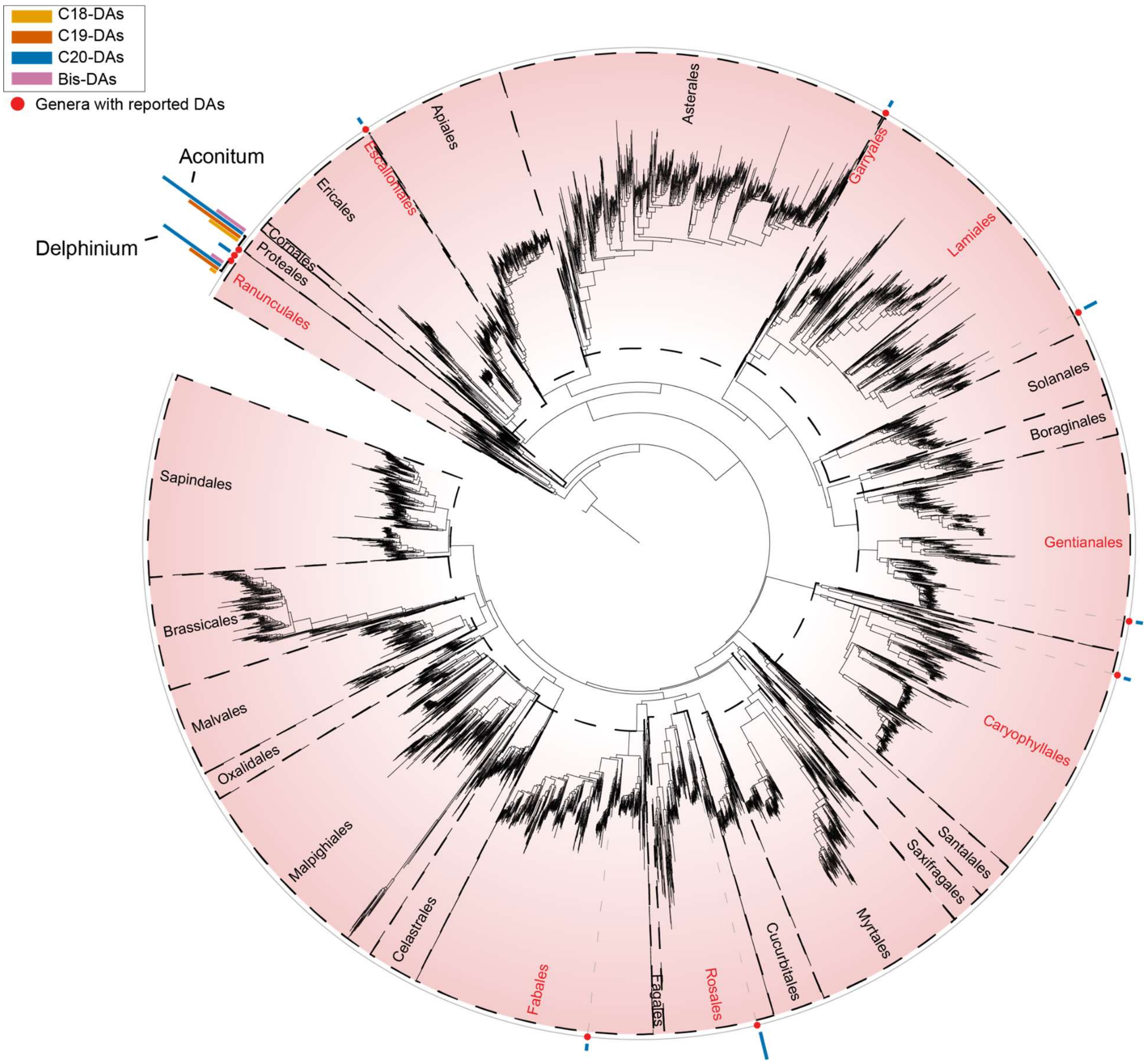

S. Figure 1. Angiosperm tree of life from Zuntini et al.[78] with mapped occurrence of diterpenoid alkaloids based on the literature review, highlighting the largest diversity of DA scaffolds observed within the Ranunculaceae family. Plant orders in the tree are separated by black dashed lines, whereas each leaf in the tree corresponds to the representative species of the genus, as described in the original publication. The original tree can be accessed at https://itol.embl.de/tree/14723112167277531728383616.

bioRxiv preprint doi: https://doi.org/10.1101/2025.05.15.654307; this version posted May 17, 2025. The copyright holder for this preprint (which was not certified by peer review) is the author/funder, who has granted bioRxiv a license to display the preprint in perpetuity. It is made available under aCC-BY 4.0 International license.

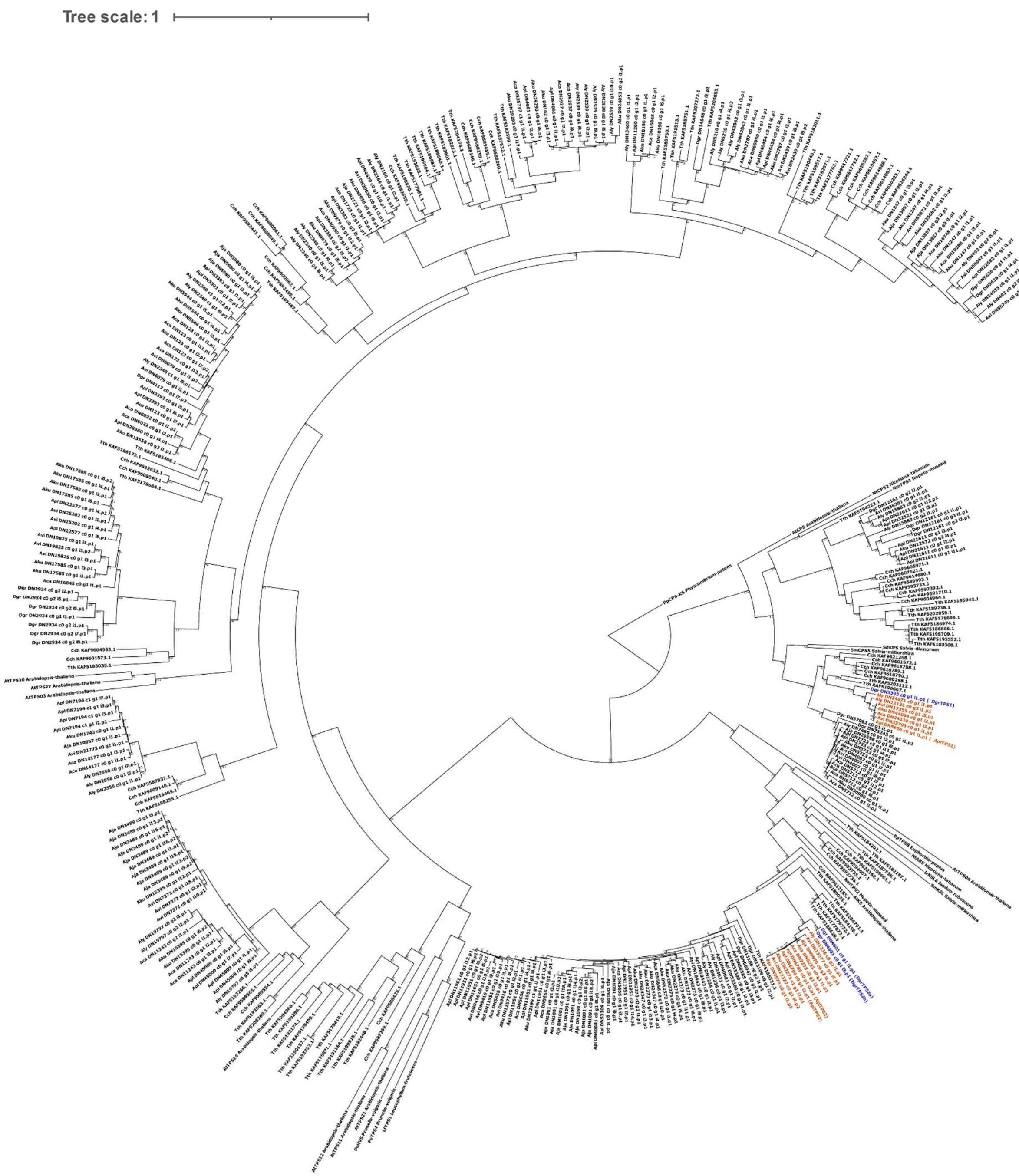

S. Figure 2. *Maximum likelihood phylogenetic tree of candidate TPSs*. This tree is an expanded view of that shown in Figure 2A. Branch lengths indicate substitutions per site and numbers at nodes represent percent support from 1,000 bootstrap replicates. A bifunctional *ent*-CPP/*ent*-kaurene synthase from *Physcomitrium patens* is used as an outgroup. Leaves in blue (*D. grandiflorum*) and orange (*Aconitum spp.*) represent sequences either characterized in this study or respective orthologs from other species. Sequences are from open reading frames of assembled transcripts, and so duplicates or assembly artifacts may be present. Abbreviations: Tth: *Thalictrum thalictroides*; Cch: *Coptis chinensis*; Dgr: *Delphinium grandiflorum*; Apl: *Aconitum plicatum*; Aly: *Aconitum lycoctonum*; Aca: *Aconitum carmichaelii*; Aja: *Aconitum japonicum*; Avi: *Aconitum vilmorinianum*; Aku: *Aconitum kusnezoffii*.

bioRxiv preprint doi: https://doi.org/10.1101/2025.05.15.654307; this version posted May 17, 2025. The copyright holder for this preprint (which was not certified by peer review) is the author/funder, who has granted bioRxiv a license to display the preprint in perpetuity. It is made available under aCC-BY 4.0 International license.

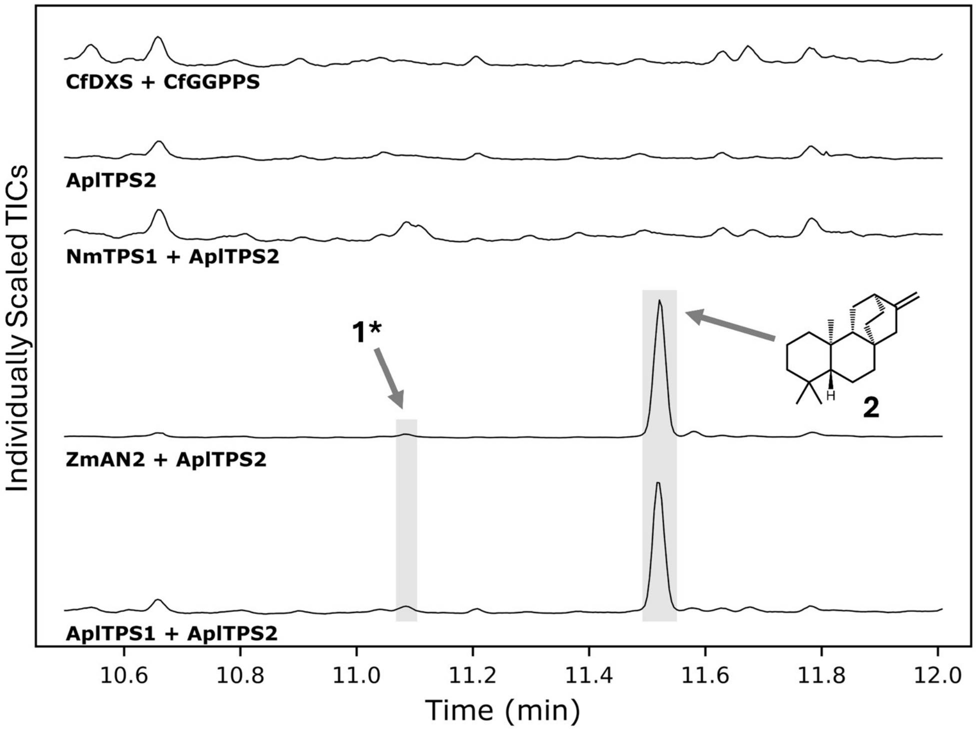

S. Figure 3. A. plicatum *TPSs testing*. GC-MS chromatograms of hexane extracts of *N. benthamiana* infiltrations. Each assay includes *Cf*DXS and *Cf*GGPPS in addition to those listed. Both *A. plicatum* TPS orthologs have the same function and enantioselectivity as *Dgr*TPS1 and *Dgr*TPS2. Retention times are not equal to other GC traces due to the use of a different GC method, as described in Methods. **1***, the dephosphorylated derivative of ent-CPP (**1**).

bioRxiv preprint doi: https://doi.org/10.1101/2025.05.15.654307; this version posted May 17, 2025. The copyright holder for this preprint (which was not certified by peer review) is the author/funder, who has granted bioRxiv a license to display the preprint in perpetuity. It is made available under aCC-BY 4.0 International license.

S. Table 1: *^1H and ^{13}C chemical shifts for* ent-*atiserene (**2**)*. $CDCl_3$ peaks were referenced to 7.26 and 77.00 ppm for ^1H and ^{13}C spectra, respectively.

^{13}C NMR	δ	^{1}H NMR
C1	39.4	0.79 (dd, J = 3.7, 13.1 Hz, 1H); 1.53 (m, 1H)
C2	18.2	1.38 (m, 1H); 1.59 (m, 1H)
C3	42.2	1.15 (m, 1H); 1.38
C4	33.1	-
C5	56.3	0.82 (dd, J = 1.7, 12.1 Hz, 1H)
C6	18.8	1.34 (m, 1H); 1.48 (ddt, *J* = 13.3, 4.6, 2.4 Hz, 1H)
C7	39.5	1.14 (m, 1H); 1.18 (m, 1H)
C8	33.5	-
C9	52.8	1.16 (m, 1H)
C10	37.7	-
C11	28.6	1.42 (m, 1H); 1.59 (m, 1H)
C12	36.6	2.24 (p, *J* = 3.0 Hz, 1H)
C13	28.7	0.99 (m, 1H); 1.94 (m, 1H)
C14	27.4	1.59 (m, 1H); 1.62 (m, 1H)
C15	48.3	1.91 (m, 1H); 2.05 (m, 1H)
C16	153.2	-
C17	104.4	4.58 (q, *J* = 2.1 Hz, 1H); 4.74 (q, *J* = 2.3 Hz, 1H)
C18	33.5	0.87 (s, 3H)
C19	21.7	0.84 (s, 3H)
C20	13.9	0.98 (s, 3H)

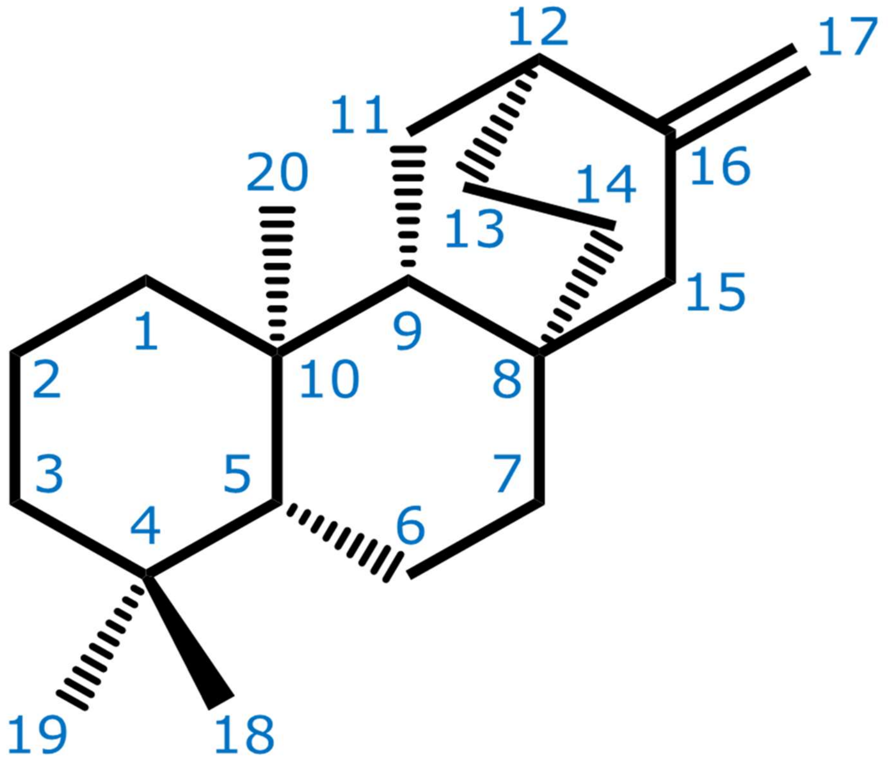

bioRxiv preprint doi: https://doi.org/10.1101/2025.05.15.654307; this version posted May 17, 2025. The copyright holder for this preprint (which was not certified by peer review) is the author/funder, who has granted bioRxiv a license to display the preprint in perpetuity. It is made available under aCC-BY 4.0 International license.

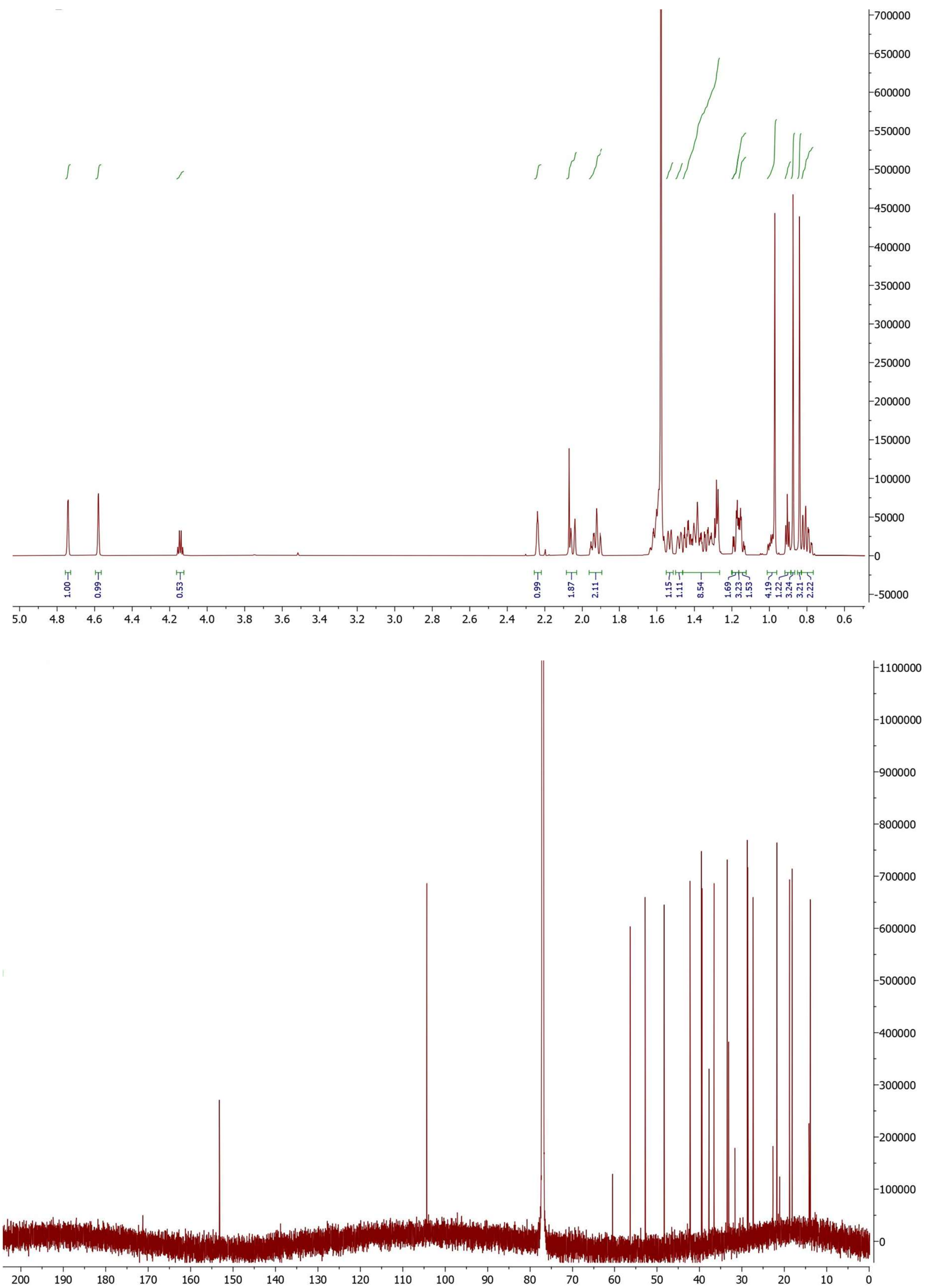

S. Figure 4: *^{1}H, ^{13}C, HSQC, H2BC, HMBC, COSY, and NOESY NMR spectra for* ent-*atiserene* (**2**).

bioRxiv preprint doi: https://doi.org/10.1101/2025.05.15.654307; this version posted May 17, 2025. The copyright holder for this preprint (which was not certified by peer review) is the author/funder, who has granted bioRxiv a license to display the preprint in perpetuity. It is made available under aCC-BY 4.0 International license.

S. Figure 4 (continued)

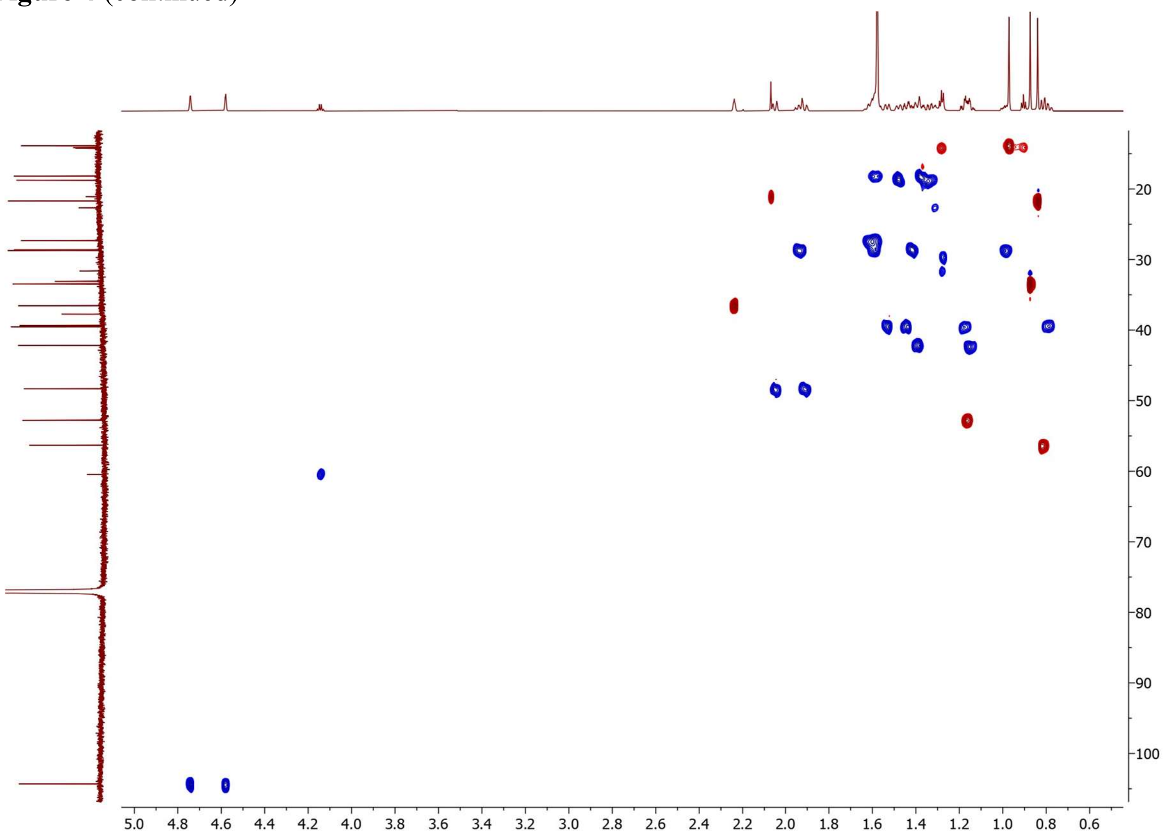

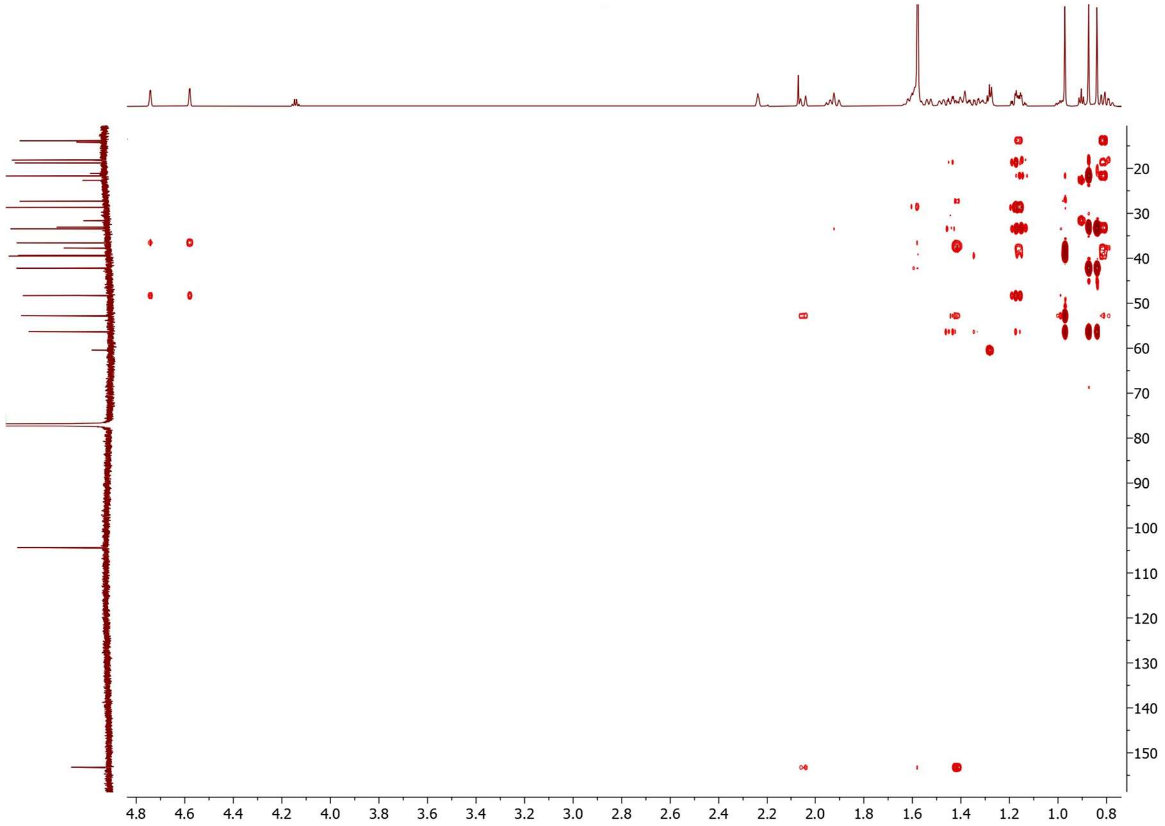

bioRxiv preprint doi: https://doi.org/10.1101/2025.05.15.654307; this version posted May 17, 2025. The copyright holder for this preprint (which was not certified by peer review) is the author/funder, who has granted bioRxiv a license to display the preprint in perpetuity. It is made available under aCC-BY 4.0 International license.

S. Figure 4 (continued)

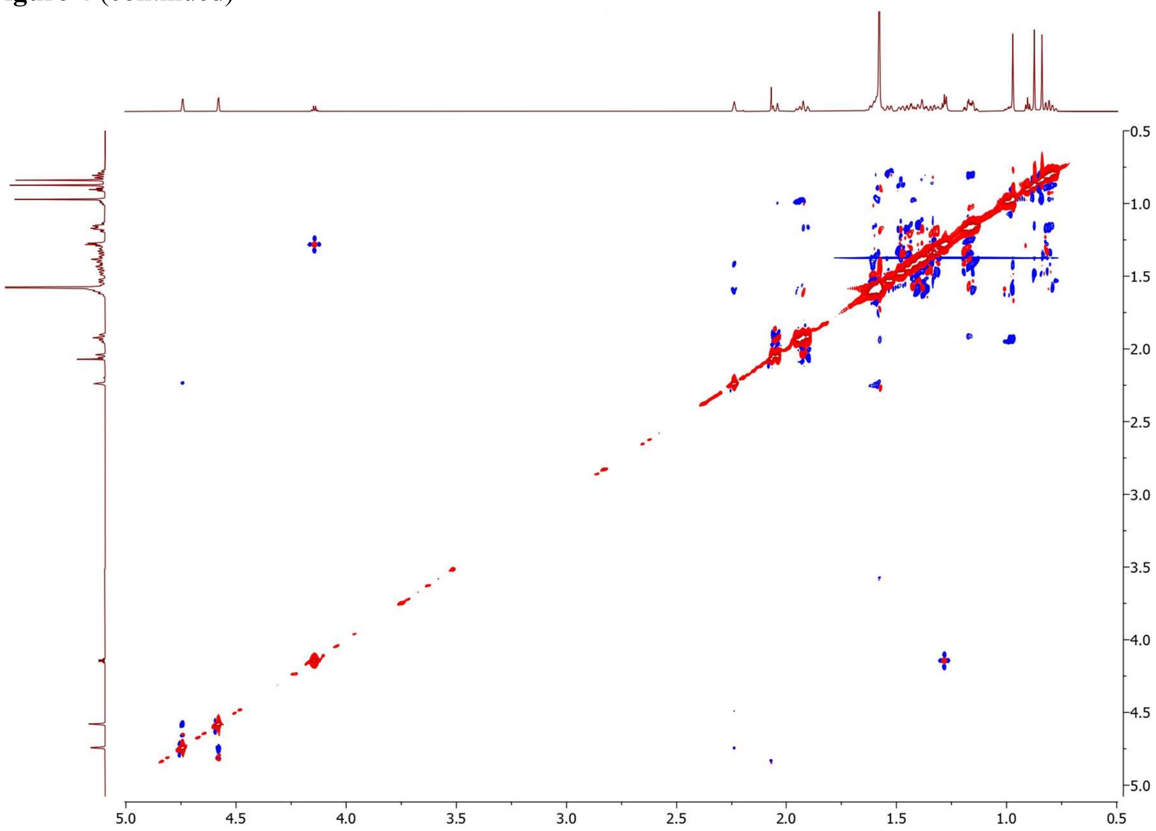

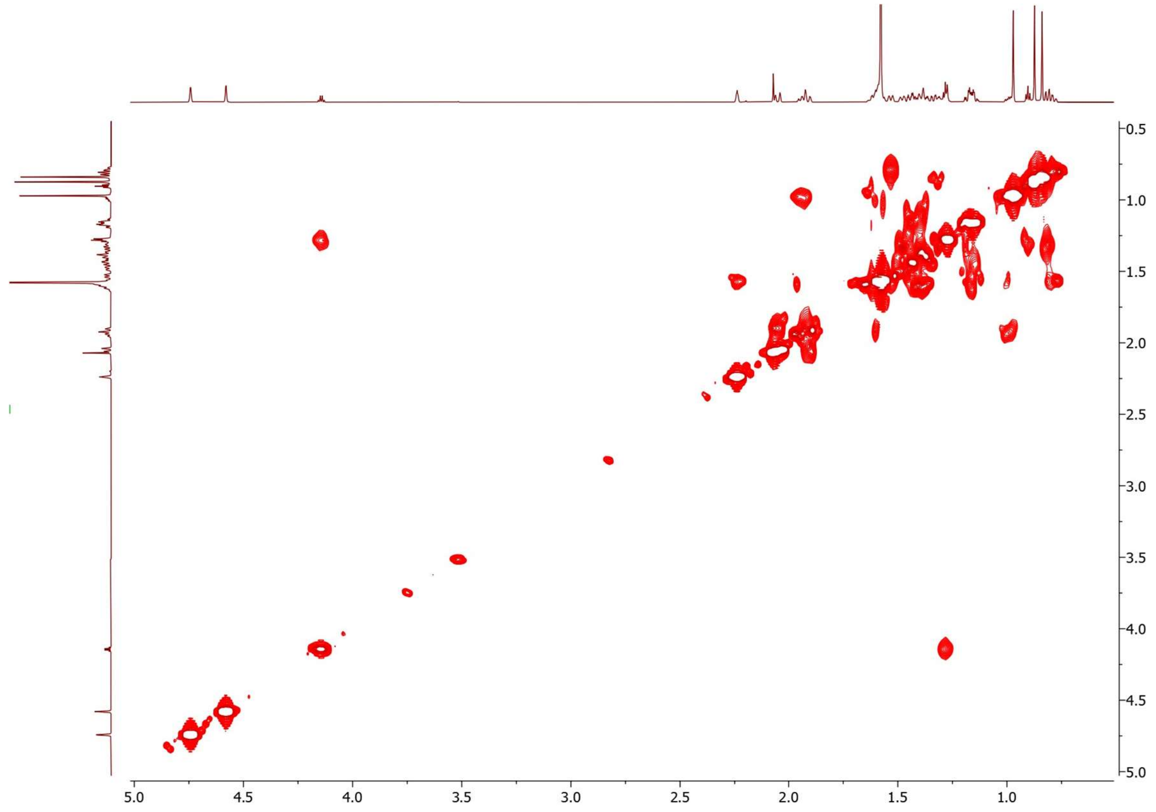

bioRxiv preprint doi: https://doi.org/10.1101/2025.05.15.654307; this version posted May 17, 2025. The copyright holder for this preprint (which was not certified by peer review) is the author/funder, who has granted bioRxiv a license to display the preprint in perpetuity. It is made available under aCC-BY 4.0 International license.

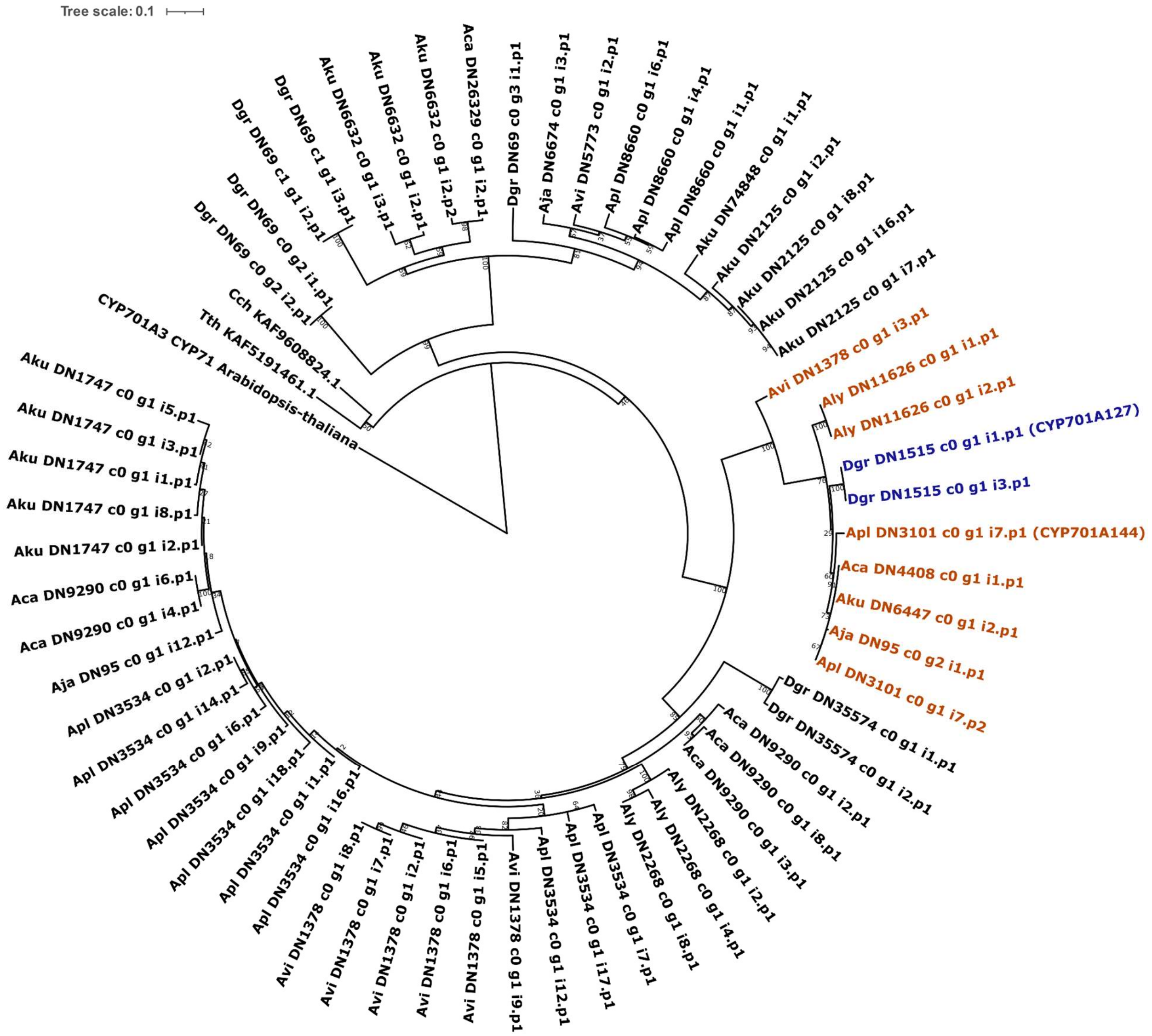

S. Figure 5: *Maximum likelihood phylogenetic tree of candidate CYPs from the CYP701 family*. This tree represents the second largest cluster within the CYP71 clan shown in the sequence similarity network in Figure 3A. Branch lengths indicate substitutions per site and numbers at nodes represent percent support from 1,000 bootstrap replicates. CYP701A3 from *Arabidopsis thaliana* is used as an outgroup. Leaves in blue (*D. grandiflorum*) and orange (*Aconitum spp.*) represent sequences either characterized in this study or respective orthologs from other species, containing CYP701A127 (*D. grandiflorum*) and CYP701A144 (*A. plicatum*). Sequences are from open reading frames of assembled transcripts, and so duplicates or assembly artifacts may be present. Abbreviations: Tth: *Thalictrum thalictroides*; Cch: *Coptis chinensis*; Dgr: *Delphinium grandiflorum*; Apl: *Aconitum plicatum*; Aly: *Aconitum lycoctonum*; Aca: *Aconitum carmichaelii*; Aja: *Aconitum japonicum*; Avi: *Aconitum vilmorinianum*; Aku: *Aconitum kusnezoffii*.

bioRxiv preprint doi: https://doi.org/10.1101/2025.05.15.654307; this version posted May 17, 2025. The copyright holder for this preprint (which was not certified by peer review) is the author/funder, who has granted bioRxiv a license to display the preprint in perpetuity. It is made available under aCC-BY 4.0 International license.

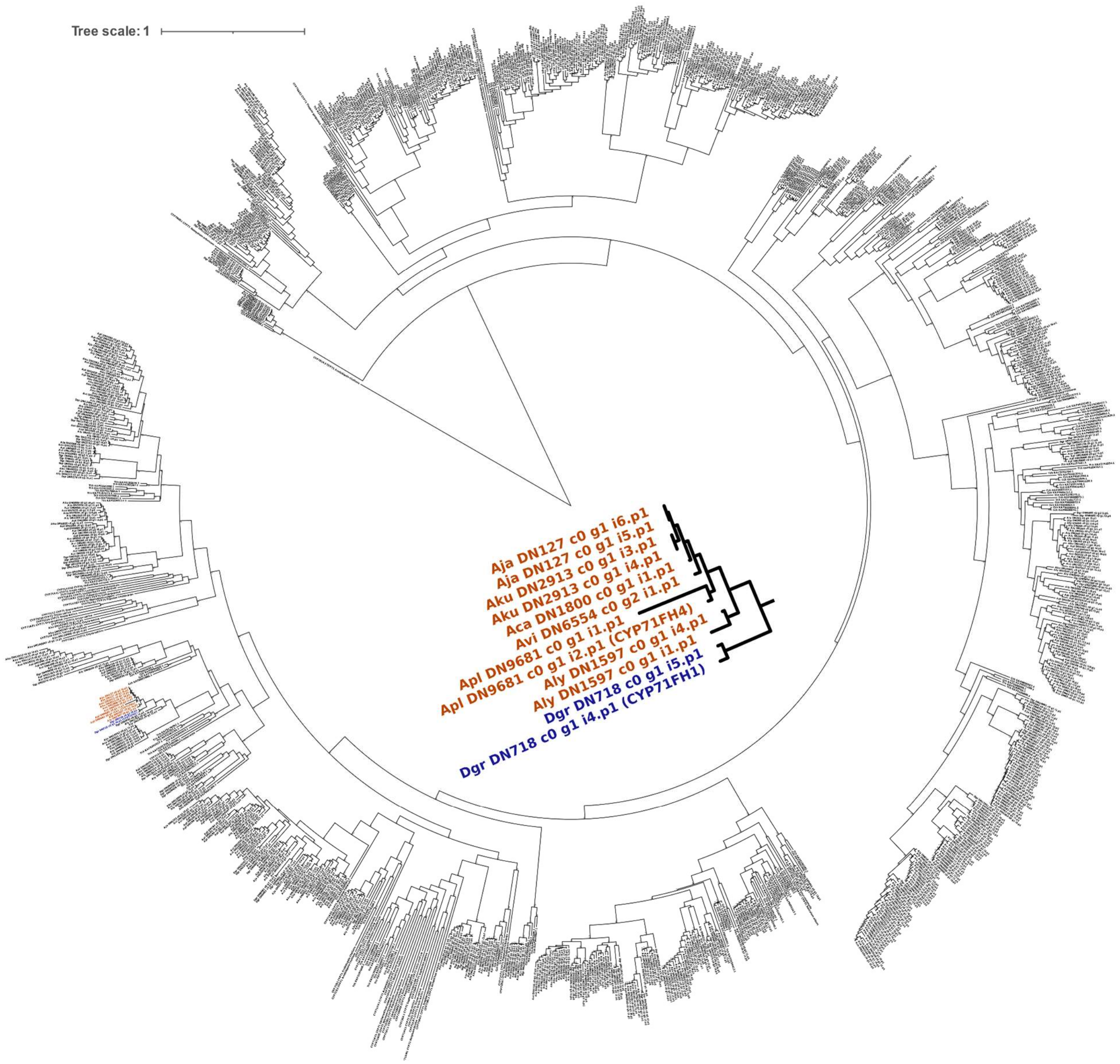

S. Figure 6: *Maximum likelihood phylogenetic tree of candidate CYPs from the CYP71 clan*. This tree represents the largest cluster within the CYP71 clan shown in the sequence similarity network in Figure 3A. Branch lengths indicate substitutions per site and numbers at nodes represent percent support from 100 bootstrap replicates. CYP701A3 from *Arabidopsis thaliana* is used as an outgroup. Leaves in blue (*D. grandiflorum*) and orange (*Aconitum spp.*) are enlarged in the center and represent sequences either characterized in this study or respective orthologs from other species, and contains CYP71FH1 (*D. grandiflorum*) and CYP71FH4 (*A. plicatum*). Sequences are from open reading frames of assembled transcripts, and so duplicates or assembly artifacts may be present. Abbreviations: Tth: *Thalictrum thalictroides*; Cch: *Coptis chinensis*; Dgr: *Delphinium grandiflorum*; Apl: *Aconitum plicatum*; Aly: *Aconitum lycoctonum*; Aca: *Aconitum carmichaelii*; Aja: *Aconitum japonicum*; Avi: *Aconitum vilmorinianum*; Aku: *Aconitum kusnezoffii*.

bioRxiv preprint doi: https://doi.org/10.1101/2025.05.15.654307; this version posted May 17, 2025. The copyright holder for this preprint (which was not certified by peer review) is the author/funder, who has granted bioRxiv a license to display the preprint in perpetuity. It is made available under aCC-BY 4.0 International license.

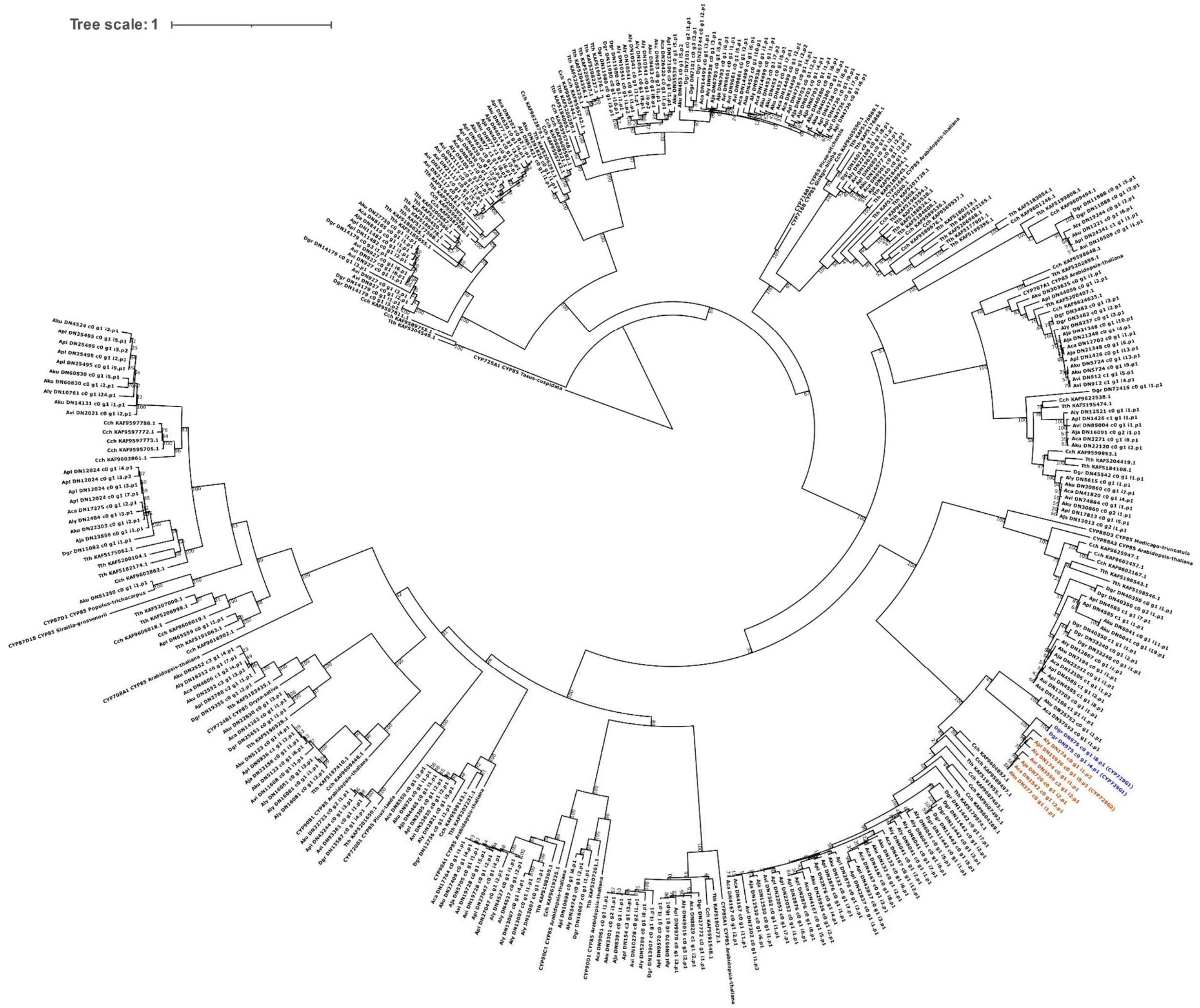

S. Figure 7: *Maximum likelihood phylogenetic tree of candidate CYPs from the CYP85 clan*. This is an expanded view of the tree shown in Figure 3A. Branch lengths indicate substitutions per site and numbers at nodes represent percent support from 1,000 bootstrap replicates. CYP701A3 from *Arabidopsis thaliana* is used as an outgroup. Leaves in blue (*D. grandiflorum*) and orange (*Aconitum spp.*) represent sequences either characterized in this study or respective orthologs from other species, containing CYP729G1 (*D. grandiflorum*) and CYP729G2 (*A. plicatum*). This highlighted region is enlarged in Figure 3A. Sequences are from open reading frames of assembled transcripts, and so duplicates or assembly artifacts may be present, and a duplicate transcript from *D. grandiflorum* was omitted for clarity within Figure 3A. Abbreviations: Tth: *Thalictrum thalictroides*; Cch: *Coptis chinensis*; Dgr: *Delphinium grandiflorum*; Apl: *Aconitum plicatum*; Aly: *Aconitum lycoctonum*; Aca: *Aconitum carmichaelii*; Aja: *Aconitum japonicum*; Avi: *Aconitum vilmorinianum*; Aku: *Aconitum kusnezoffii*.

bioRxiv preprint doi: https://doi.org/10.1101/2025.05.15.654307; this version posted May 17, 2025. The copyright holder for this preprint (which was not certified by peer review) is the author/funder, who has granted bioRxiv a license to display the preprint in perpetuity. It is made available under aCC-BY 4.0 International license.

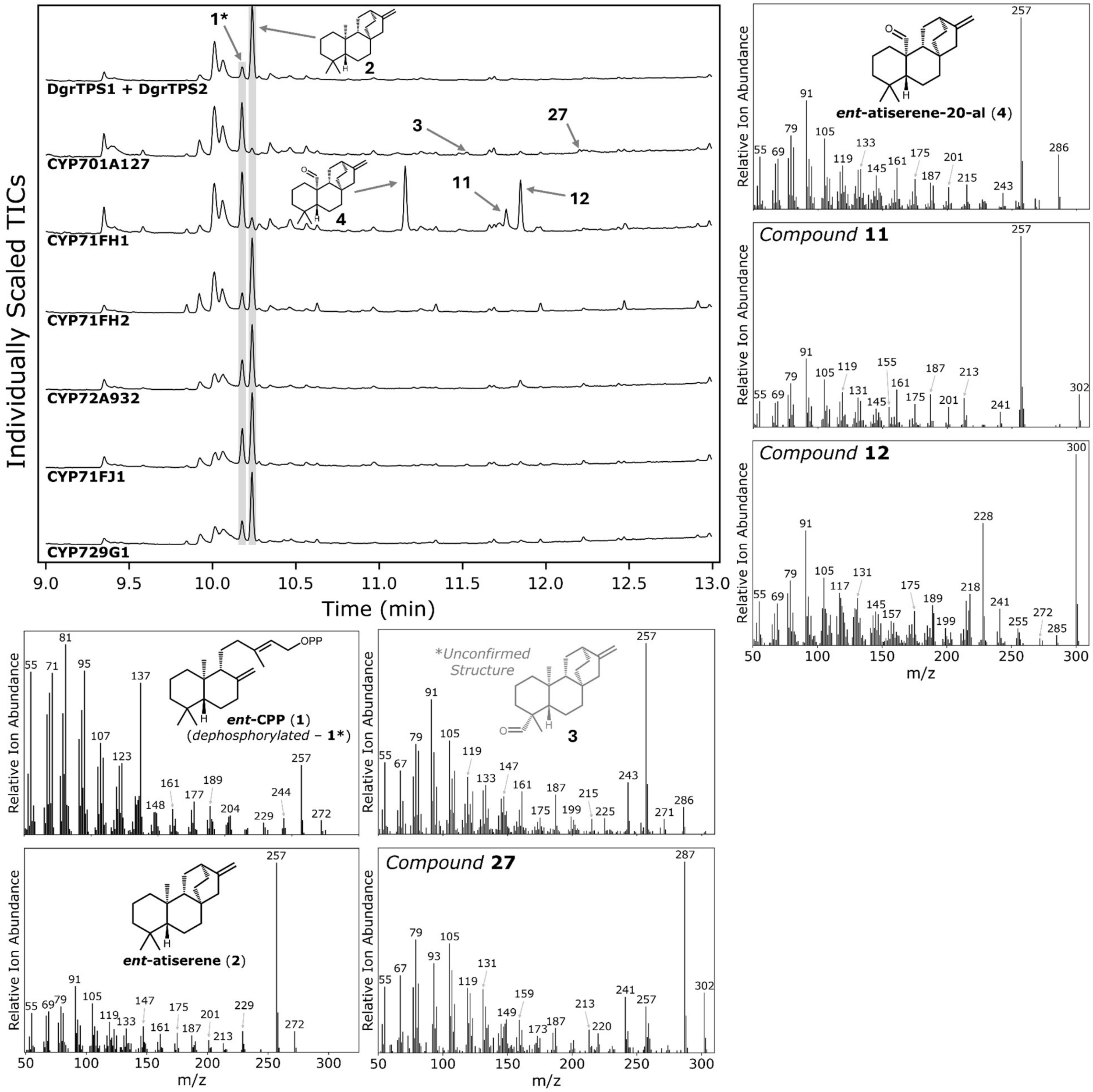

S. Figure 8: *Initial screening of six CYPs with both TPSs*. (Top Left) GC-MS chromatograms of EtOAc extracts of *N. benthamiana* infiltrations. Each assay includes *Cf*DXS, *Cf*GGPPS, *Dgr*TPS1, and *Dgr*TPS2 in addition to those listed. Compound **27** was not observed consistently across assays, likely due to low abundance. (Bottom Left) Mass spectra for compounds made through coexpression of *Dgr*TPS1 and *Dgr*TPS2. (Bottom Middle) Mass spectra for compounds made through coexpression of CYP701A127 with previous pathway steps. (Right) Mass spectra for compounds made through coexpression of CYP71FH1 with previous pathway steps.

bioRxiv preprint doi: https://doi.org/10.1101/2025.05.15.654307; this version posted May 17, 2025. The copyright holder for this preprint (which was not certified by peer review) is the author/funder, who has granted bioRxiv a license to display the preprint in perpetuity. It is made available under aCC-BY 4.0 International license.

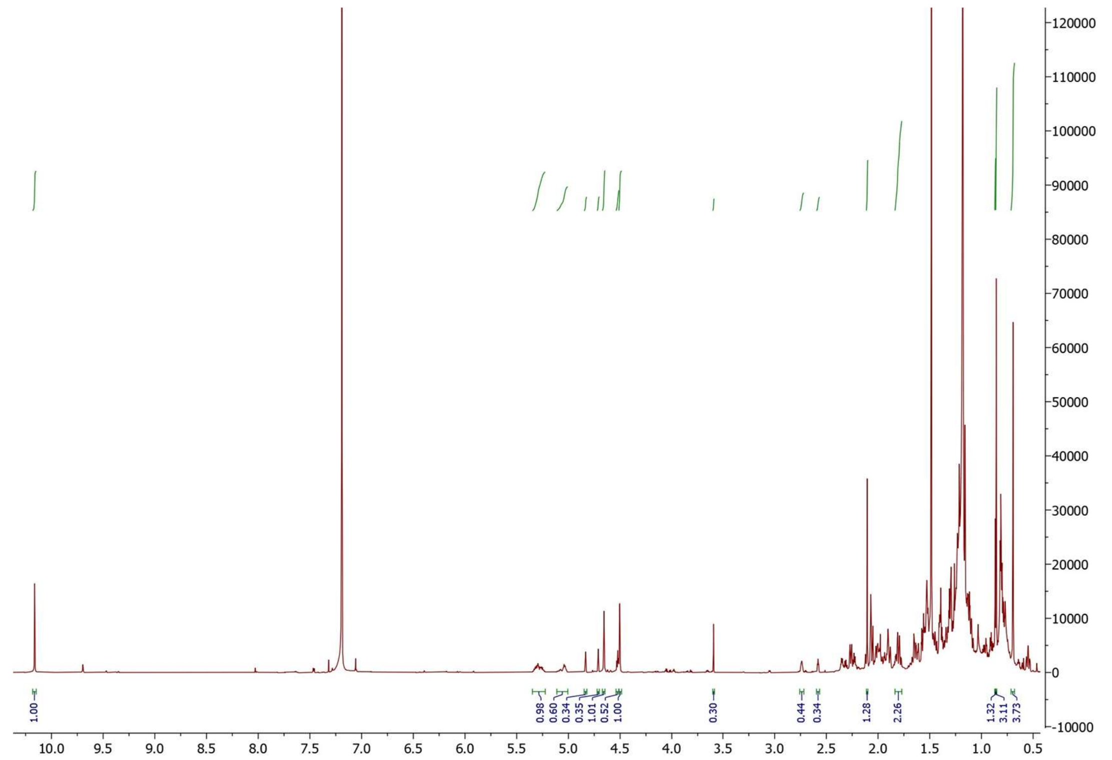

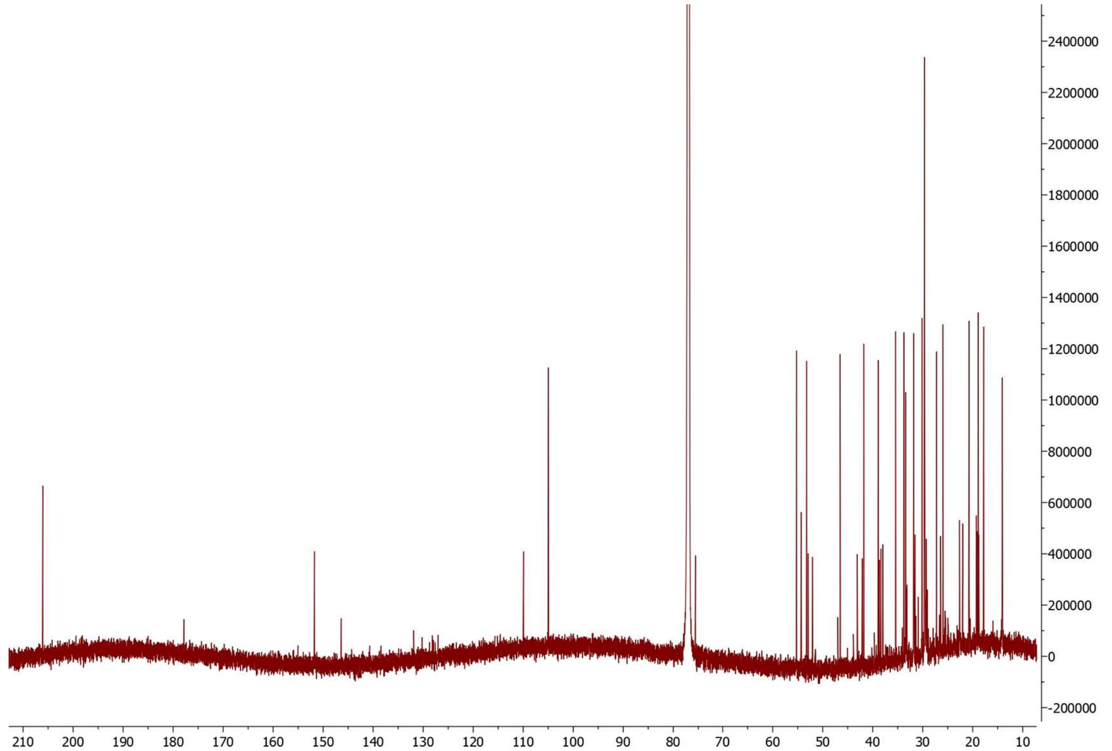

S. Figure 9: *^{1}H, ^{13}C, HSQC, H2BC, HMBC, NOESY, and COSY NMR spectra of* ent-*atiserene-20-al (**4**)*. Aldehyde peak is present in ^{1}H spectrum at 10.16 ppm, which has the same integration value as terminal alkene protons (4.50 and 4.66 ppm). This product was not completely purified from **12** (peaks at 4.71 and 4.83 ppm are likely terminal alkene protons for **12**).

bioRxiv preprint doi: https://doi.org/10.1101/2025.05.15.654307; this version posted May 17, 2025. The copyright holder for this preprint (which was not certified by peer review) is the author/funder, who has granted bioRxiv a license to display the preprint in perpetuity. It is made available under aCC-BY 4.0 International license.

S. Figure 9 (continued)

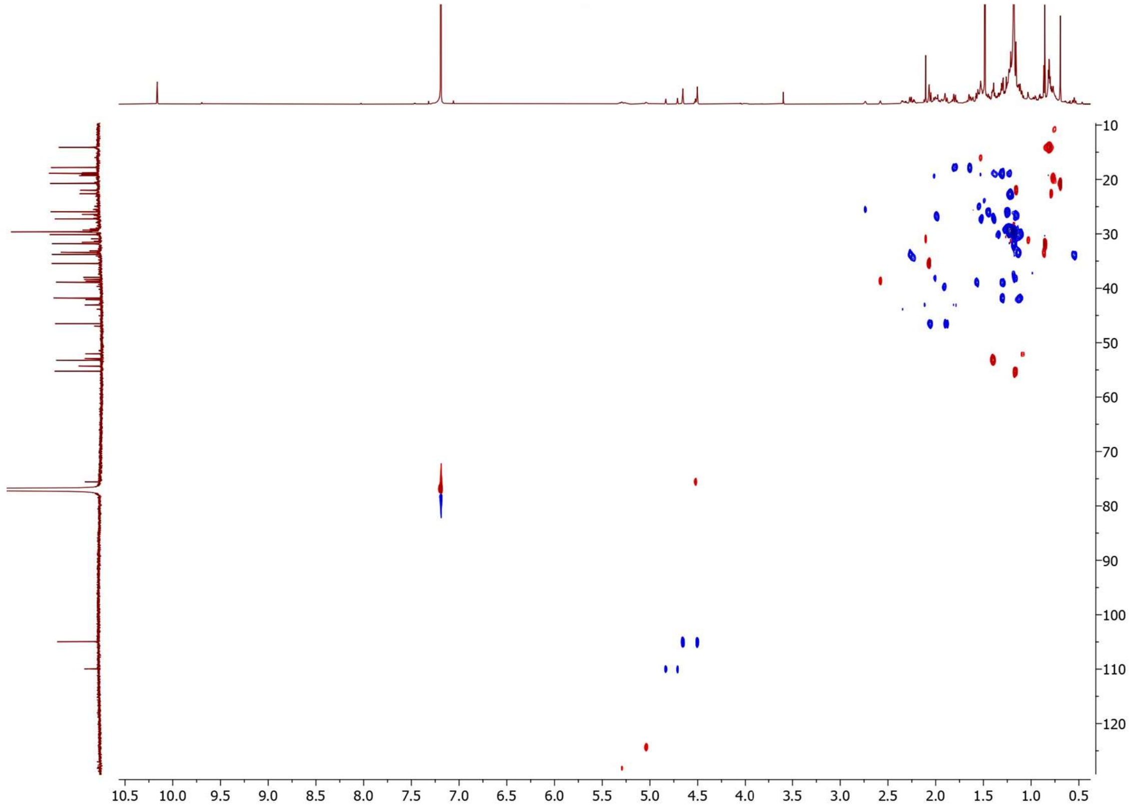

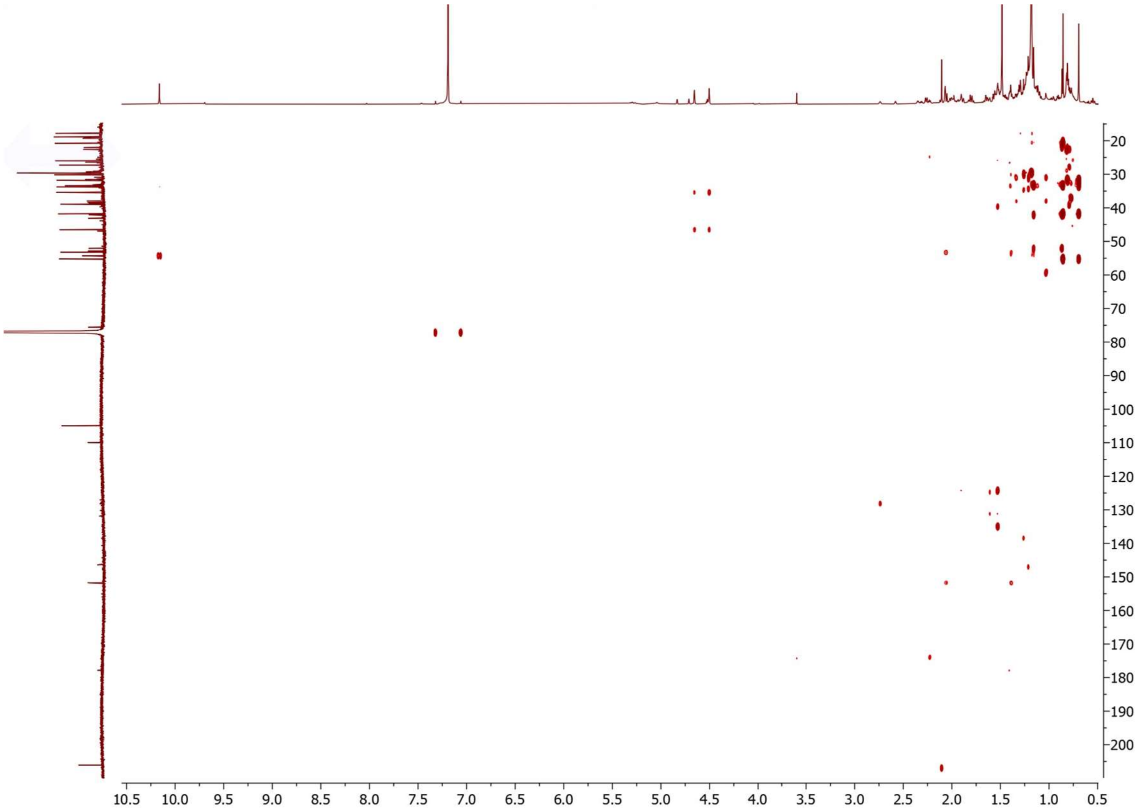

bioRxiv preprint doi: https://doi.org/10.1101/2025.05.15.654307; this version posted May 17, 2025. The copyright holder for this preprint (which was not certified by peer review) is the author/funder, who has granted bioRxiv a license to display the preprint in perpetuity. It is made available under aCC-BY 4.0 International license.

S. Figure 9 (continued)

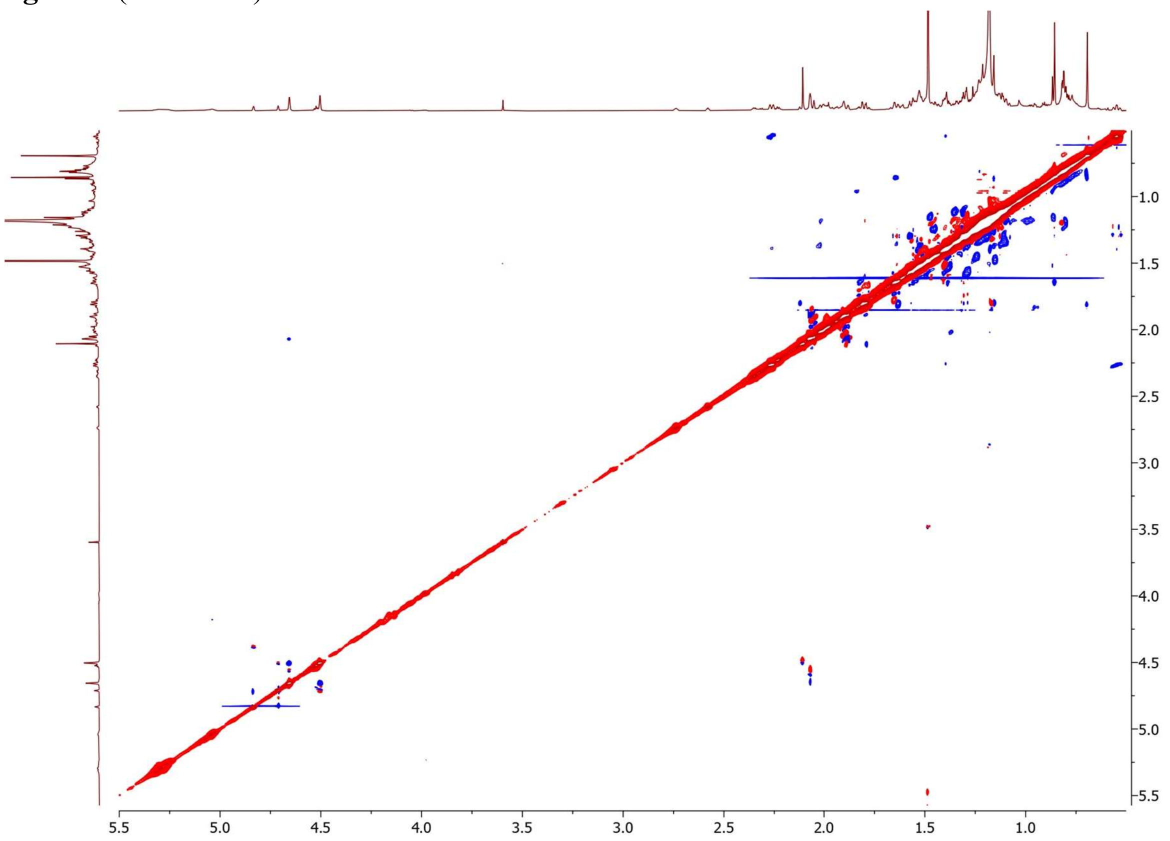

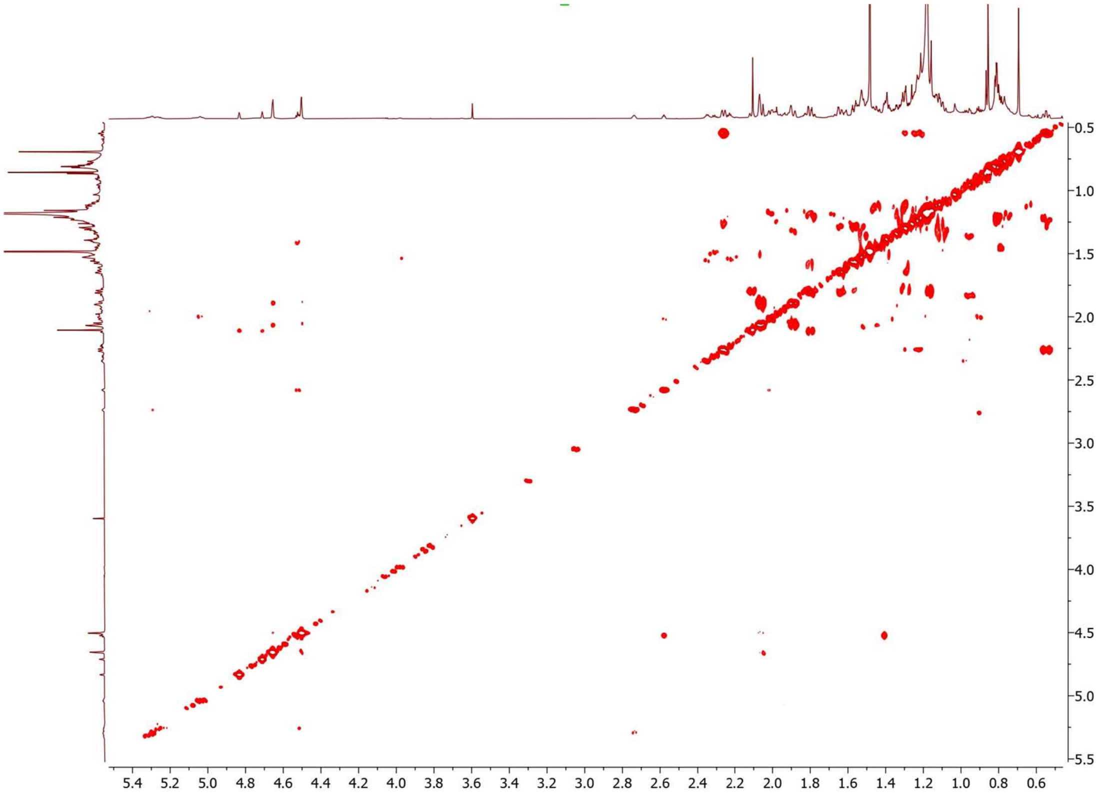

bioRxiv preprint doi: https://doi.org/10.1101/2025.05.15.654307; this version posted May 17, 2025. The copyright holder for this preprint (which was not certified by peer review) is the author/funder, who has granted bioRxiv a license to display the preprint in perpetuity. It is made available under aCC-BY 4.0 International license.

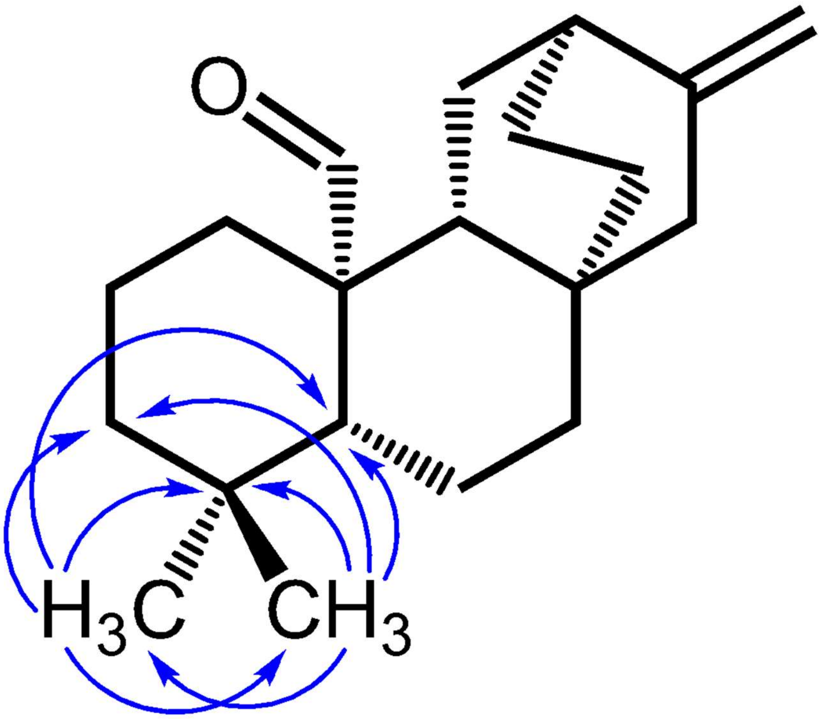

S. Figure 10: *Select HMBC correlations for* ent-*atiserene-20-al (4)*. Correlations drawn show methyl groups for carbons 18 and 19 are retained following conversion of *ent*-atiserene (**2**) by CYP71FH1.

bioRxiv preprint doi: https://doi.org/10.1101/2025.05.15.654307; this version posted May 17, 2025. The copyright holder for this preprint (which was not certified by peer review) is the author/funder, who has granted bioRxiv a license to display the preprint in perpetuity. It is made available under aCC-BY 4.0 International license.

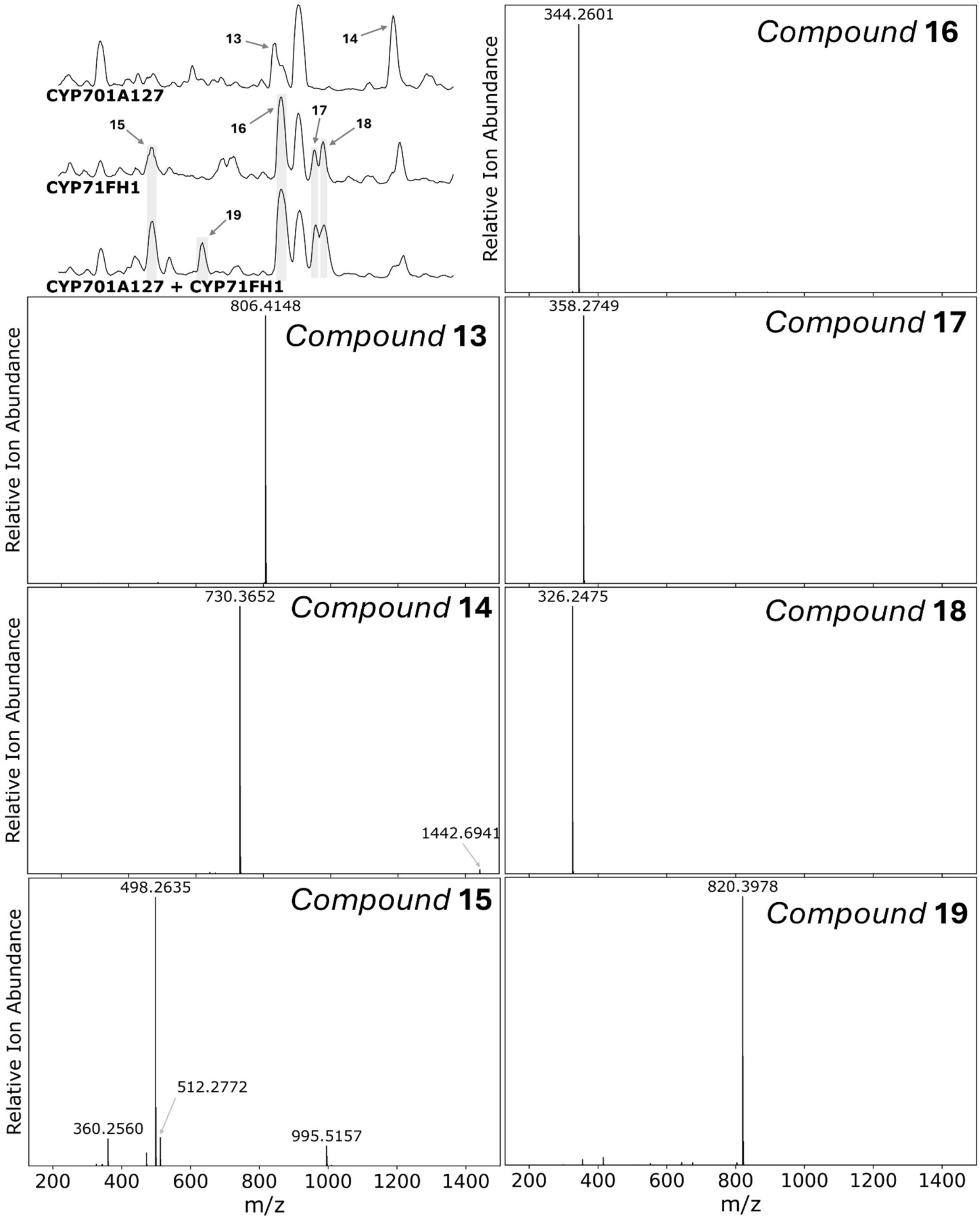

S. Figure 11: *Mass spectra for all compounds shown in Figure 3C in the main text for CYP701A127 and CYP71FH1*. Mass spectra were recorded in positive ion mode. Relevant portion of Figure 3C is shown in the top left.

bioRxiv preprint doi: https://doi.org/10.1101/2025.05.15.654307; this version posted May 17, 2025. The copyright holder for this preprint (which was not certified by peer review) is the author/funder, who has granted bioRxiv a license to display the preprint in perpetuity. It is made available under aCC-BY 4.0 International license.

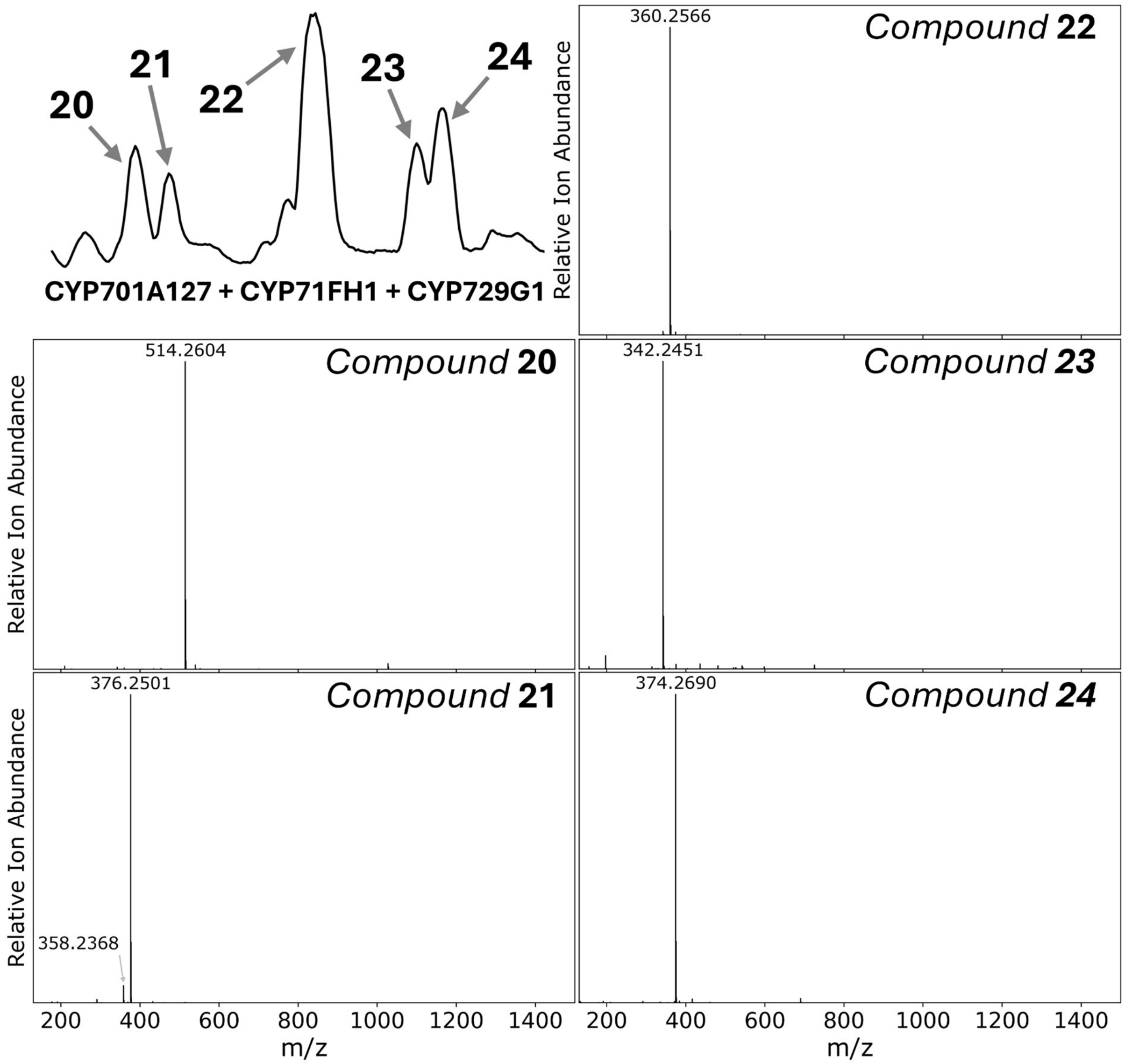

S. Figure 12: *Mass spectra for all compounds shown in Figure 3C in the main text for coexpression of each CYP together*. Mass spectra were recorded in positive ion mode. Relevant portion of Figure 3C is shown in the top left.

bioRxiv preprint doi: https://doi.org/10.1101/2025.05.15.654307; this version posted May 17, 2025. The copyright holder for this preprint (which was not certified by peer review) is the author/funder, who has granted bioRxiv a license to display the preprint in perpetuity. It is made available under aCC-BY 4.0 International license.

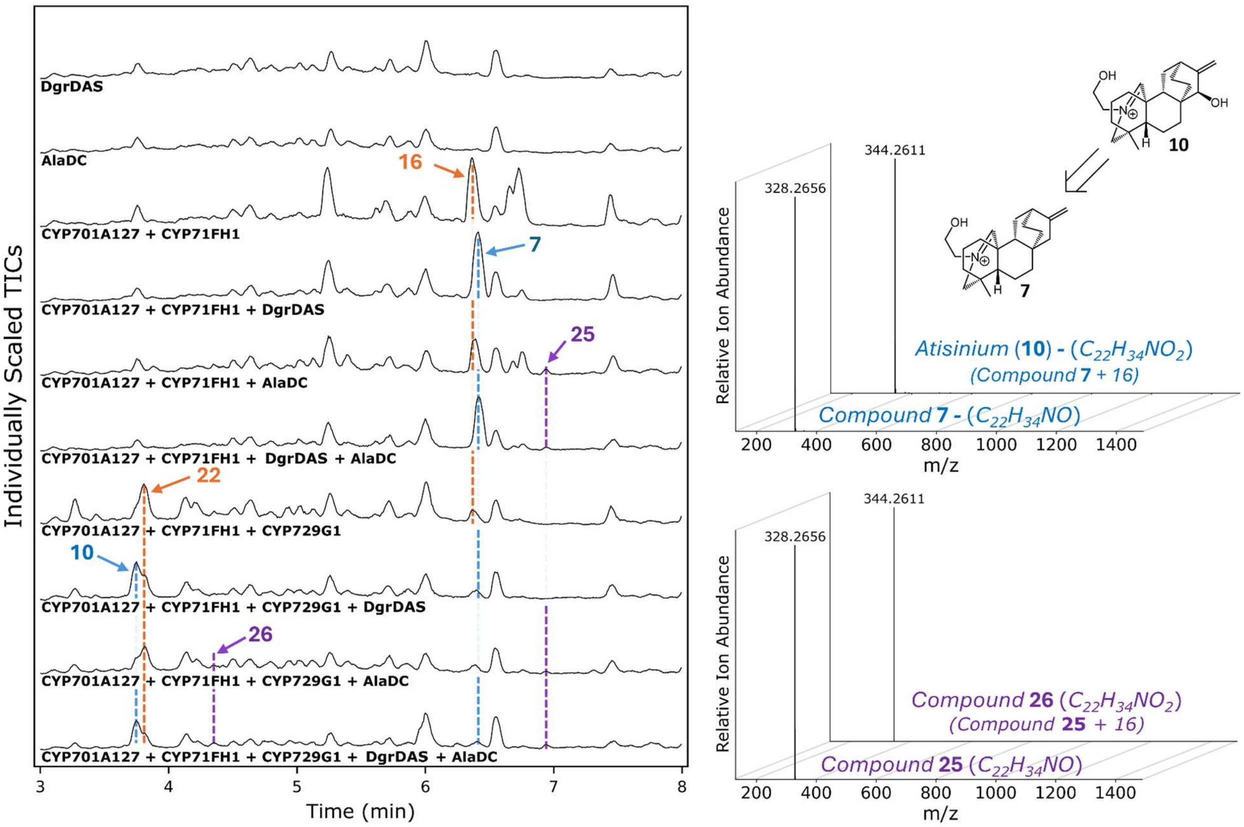

S. Figure 13: *Initial testing to supplement the pathway with ethylamine through AlaDC*. LC-MS chromatograms of 80% MeOH extracts of *N. benthamiana* infiltrations. Each assay includes *Cf*DXS, *Cf*GGPPS, *Dgr*TPS1, and *Dgr*TPS2 in addition to those listed. An alanine decarboxylase (AlaDC) was introduced to preceding pathway genes as an initial test to see how product profile changes with a presumed increase in supply of ethylamine. Only a trace amount of new product was observed, even when combined with *Dgr*DAS, and these products did not overlap with *Dgr*DAS's products. Products traced in orange are products of CYPs alone, in blue are products that form with addition of *Dgr*DAS, and in purple are products that form with addition of AlaDC. Note that only select products are highlighted in this figure. A potential structure for **7** is drawn based on the difference in observed molecular weight of 16 m/z between atisinium (**10**) and **7**, and the conditions which result in this additional product involving an additional CYP (CYP729G1).

bioRxiv preprint doi: https://doi.org/10.1101/2025.05.15.654307; this version posted May 17, 2025. The copyright holder for this preprint (which was not certified by peer review) is the author/funder, who has granted bioRxiv a license to display the preprint in perpetuity. It is made available under aCC-BY 4.0 International license.

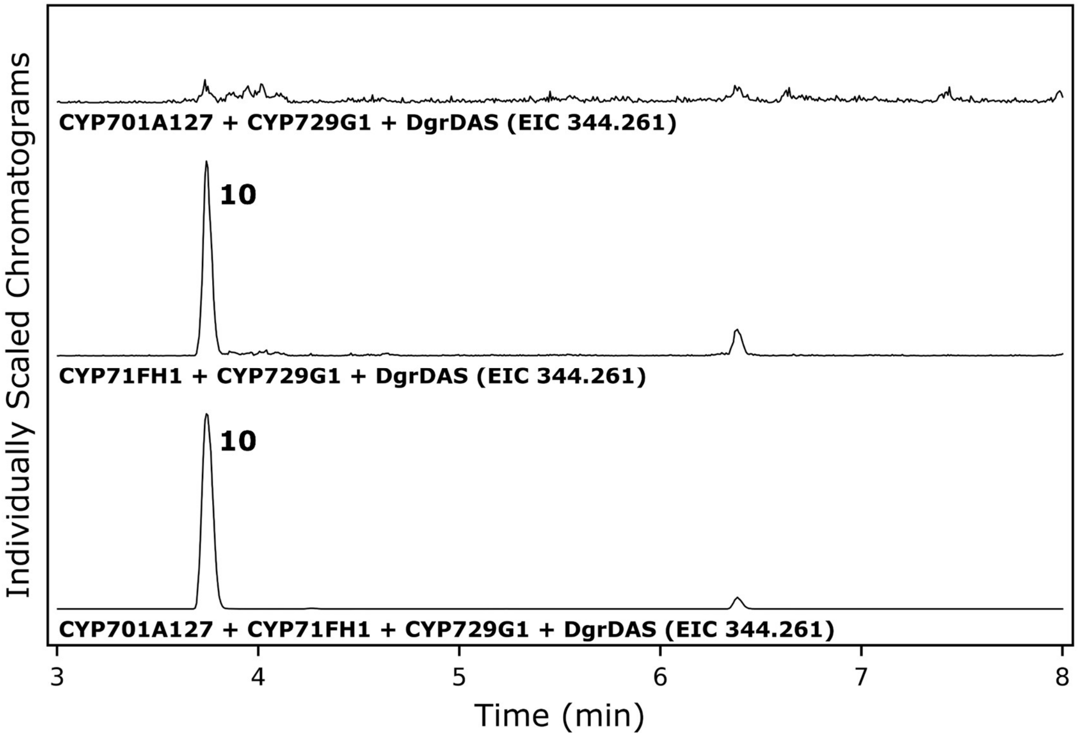

S. Figure 14: *CYP71FH1 complements a lack of CYP701A127 in atisinium (**10**) formation.* LC-MS chromatograms of 80% MeOH extracts of *N. benthamiana* infiltrations. Each assay includes *Cf*DXS, *Cf*GGPPS, *Dgr*TPS1, and *Dgr*TPS2 in addition to those listed.

bioRxiv preprint doi: https://doi.org/10.1101/2025.05.15.654307; this version posted May 17, 2025. The copyright holder for this preprint (which was not certified by peer review) is the author/funder, who has granted bioRxiv a license to display the preprint in perpetuity. It is made available under aCC-BY 4.0 International license.

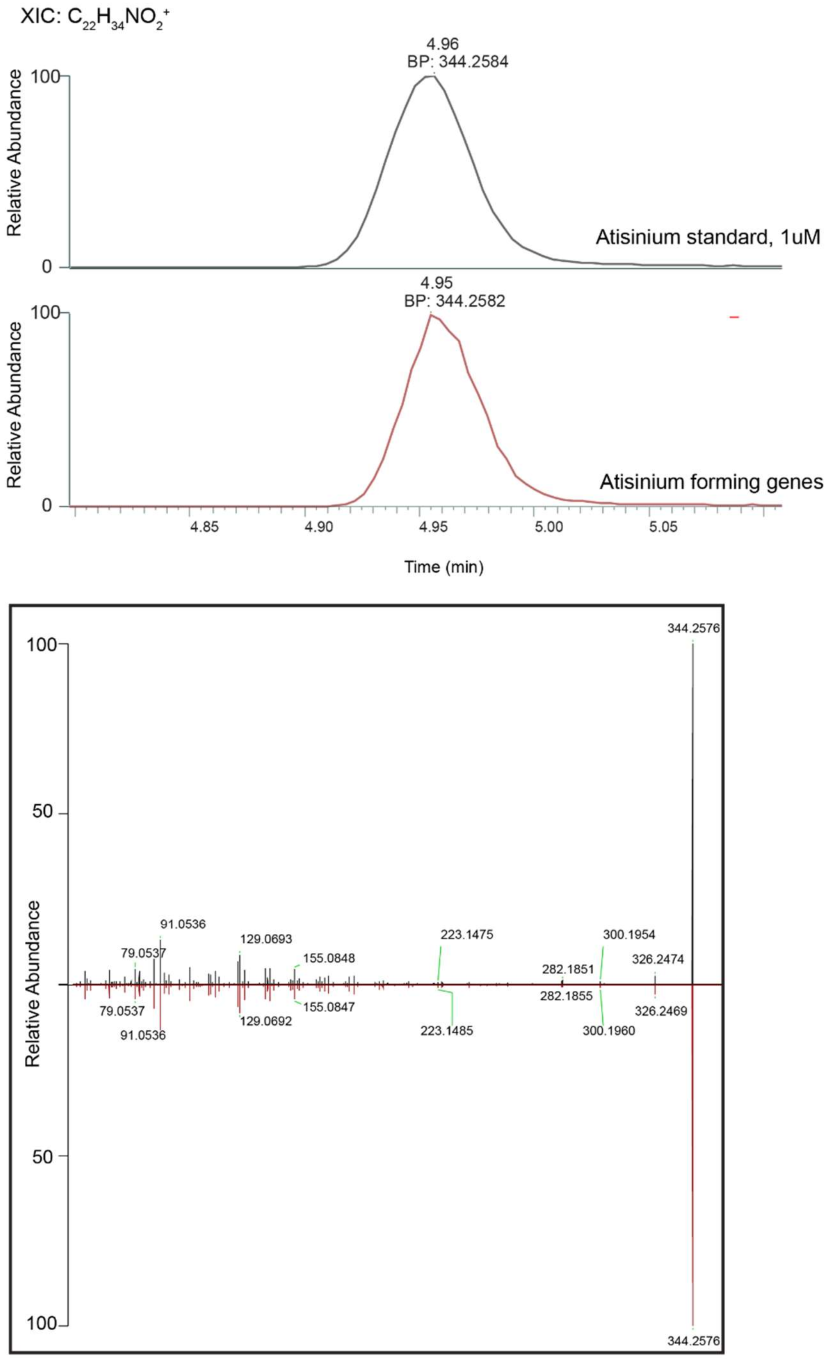

S. Figure 15: Confirmation of atisinium formation by infiltrating characterized genes in *N. benthamiana* (*Apl*TPS1, *Apl*TPS2, CYP71FH4, CYP701A144, CYP729G2, *Apl*DAS) and comparing the retention time (upper panel) and MS/MS spectra (lower panel) with atisinium standard.

bioRxiv preprint doi: https://doi.org/10.1101/2025.05.15.654307; this version posted May 17, 2025. The copyright holder for this preprint (which was not certified by peer review) is the author/funder, who has granted bioRxiv a license to display the preprint in perpetuity. It is made available under aCC-BY 4.0 International license.

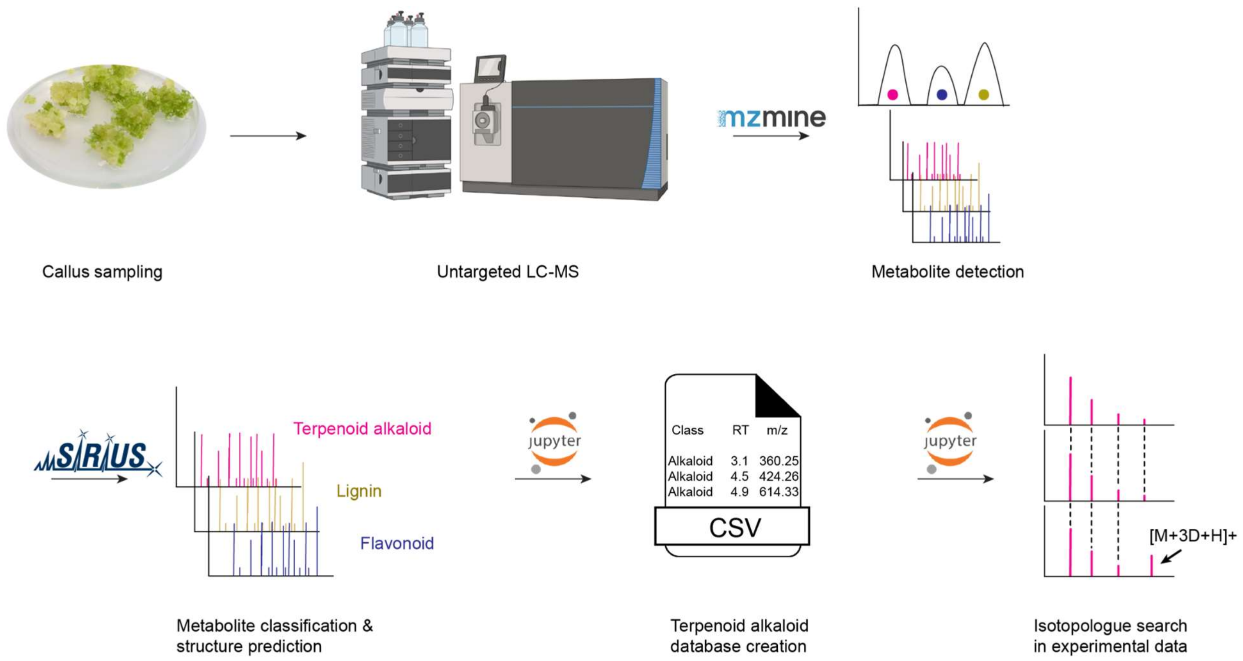

S. Figure 16. Workflow involved in identification of isotopologues from isotopically-labelled substrate feeding of *A. plicatum* callus cultures.
